# ROS/mtROS promotes TNTs formation via the PI3K/AKT/mTOR pathway to protect against mitochondrial damages in glial cells induced by engineered nanomaterials

**DOI:** 10.1186/s12989-024-00562-0

**Published:** 2024-01-15

**Authors:** Xinpei Lin, Wei Wang, Xiangyu Chang, Cheng Chen, Zhenkun Guo, Guangxia Yu, Wenya Shao, Siying Wu, Qunwei Zhang, Fuli Zheng, Huangyuan Li

**Affiliations:** 1 Department of Preventive Medicine, School of Public Health, Fujian Medical University, Fuzhou, 350122 Fujian Province China; 2 Fujian Provincial Key Laboratory of Molecular Neurology, Institute of Neuroscience, Fujian Medical University, Fuzhou, 350004 Fujian Province China; 3 The Key Laboratory of Environment and Health, School of Public Health, Fujian Medical University, Fuzhou, 350122 Fujian Province China; 4 Department of Epidemiology and Health Statistics, School of Public Health, Fujian Medical University, Fuzhou, 350122 Fujian Province China; 5 Department of Epidemiology and Population Health, School of Public Health and Information Sciences, University of Louisville, 485 E. Gray Street, Louisville, USA

**Keywords:** Cobalt nanoparticles, Titanium dioxide nanoparticles, Multi-walled carbon nanotubes, Tunneling nanotubes, ROS, mtROS, PI3K, AKT, mTOR

## Abstract

**Background:**

As the demand and application of engineered nanomaterials have increased, their potential toxicity to the central nervous system has drawn increasing attention. Tunneling nanotubes (TNTs) are novel cell–cell communication that plays a crucial role in pathology and physiology. However, the relationship between TNTs and nanomaterials neurotoxicity remains unclear. Here, three types of commonly used engineered nanomaterials, namely cobalt nanoparticles (CoNPs), titanium dioxide nanoparticles (TiO_2_NPs), and multi-walled carbon nanotubes (MWCNTs), were selected to address this limitation.

**Results:**

After the complete characterization of the nanomaterials, the induction of TNTs formation with all of the nanomaterials was observed using high-content screening system and confocal microscopy in both primary astrocytes and U251 cells. It was further revealed that TNT formation protected against nanomaterial-induced neurotoxicity due to cell apoptosis and disrupted ATP production. We then determined the mechanism underlying the protective role of TNTs. Since oxidative stress is a common mechanism in nanotoxicity, we first observed a significant increase in total and mitochondrial reactive oxygen species (namely ROS, mtROS), causing mitochondrial damage. Moreover, pretreatment of U251 cells with either the ROS scavenger N-acetylcysteine or the mtROS scavenger mitoquinone attenuated nanomaterial-induced neurotoxicity and TNTs generation, suggesting a central role of ROS in nanomaterials-induced TNTs formation. Furthermore, a vigorous downstream pathway of ROS, the PI3K/AKT/mTOR pathway, was found to be actively involved in nanomaterials-promoted TNTs development, which was abolished by LY294002, Perifosine and Rapamycin, inhibitors of PI3K, AKT, and mTOR, respectively. Finally, western blot analysis demonstrated that ROS and mtROS scavengers suppressed the PI3K/AKT/mTOR pathway, which abrogated TNTs formation.

**Conclusion:**

Despite their biophysical properties, various types of nanomaterials promote TNTs formation and mitochondrial transfer, preventing cell apoptosis and disrupting ATP production induced by nanomaterials. ROS/mtROS and the activation of the downstream PI3K/AKT/mTOR pathway are common mechanisms to regulate TNTs formation and mitochondrial transfer. Our study reveals that engineered nanomaterials share the same molecular mechanism of TNTs formation and intercellular mitochondrial transfer, and the proposed adverse outcome pathway contributes to a better understanding of the intercellular protection mechanism against nanomaterials-induced neurotoxicity.

**Graphical abstract:**

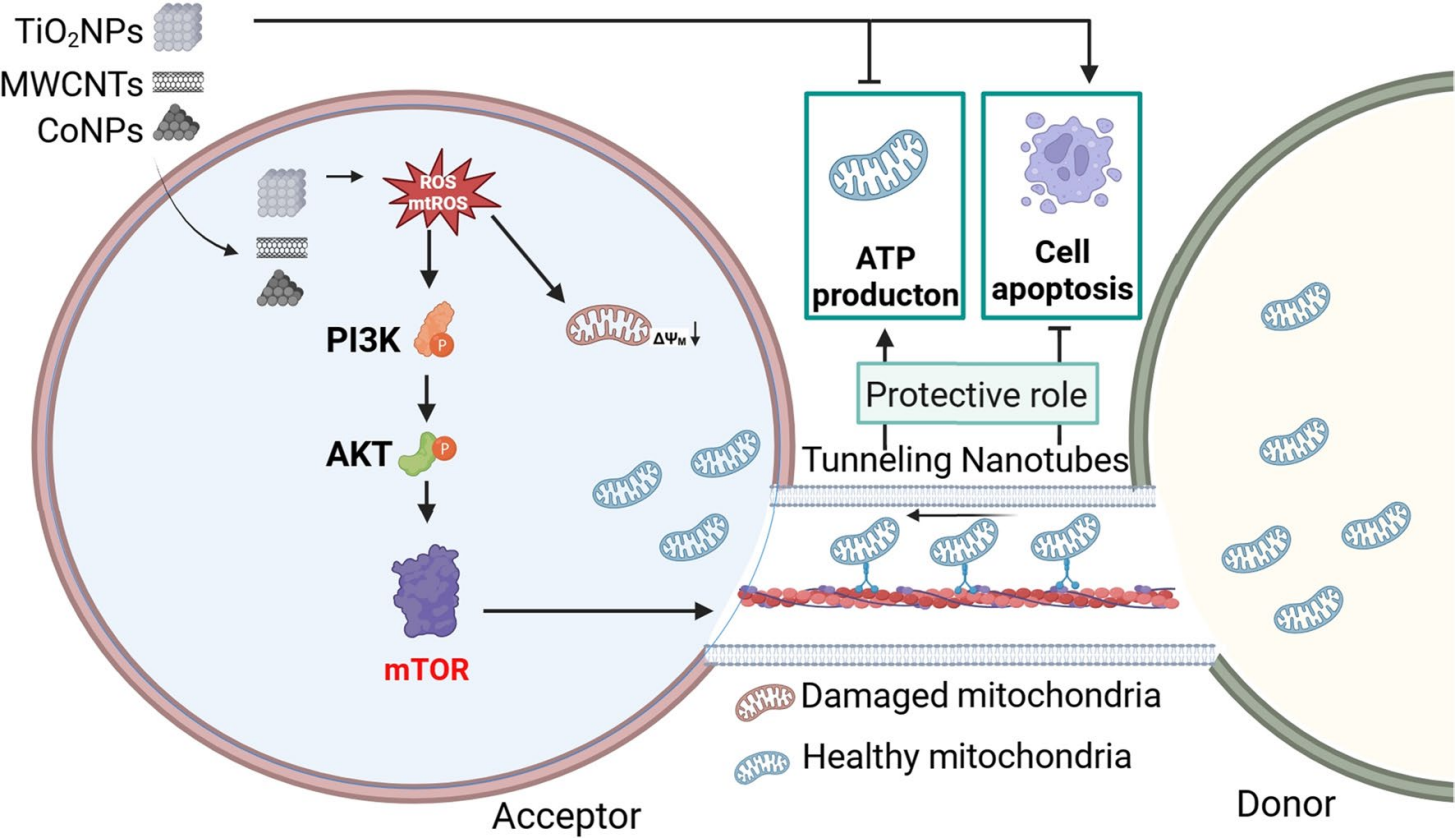

**Supplementary Information:**

The online version contains supplementary material available at 10.1186/s12989-024-00562-0.

## Introduction

Engineered nanomaterials are a broad class of materials developed to have at least one dimension between 1 and 100 nm, and offer unique, size-dependent properties not exhibited by their bulk counterparts [1]. The global nanomaterials market size was valued at USD 10.88 billion in 2022 and is expected to grow at a compound annual growth rate (CAGR) of 14.8% from 2023 to 2030, in which titanium and carbon nanotubes are the most used in the market [2]. Moreover, with the explosive global production and sales of new electric vehicles, cobalt use will continue a bullish trend with an expected CAGR of at least 30% by 2025 [3]. With the increasing application of engineered nanomaterials, their dissemination into the environment will adversely affect human health, including impairment of the central nervous system.

Cobalt nanoparticles (CoNPs), titanium dioxide nanoparticles (TiO_2_NPs), and multi-walled carbon nanotubes (MWCNTs) are widely designed and manufactured in biomedicine, electronics, energy storage, textiles, and cosmetics, as well as high-performance intermediates such as coatings and composites for aerospace, automobiles, and construction [4]. CoNPs have been applied in pigments, catalysis, sensors, electrochemistry, magnetism, and energy storage owing to their unique physical properties [5], TiO_2_NPs have been applied in nanodermatology and nanocosmetology [6] and MWCNTs have been widely used in the medical field as carriers of drug delivery [7]. However, the toxicity of nanomaterials is largely dependent on their biophysical properties, including their size, surface charge, and aggregation state [8]. Therefore, it is necessary for people to compare the toxicity of different nanomaterials to understand the influence of physical and chemical properties of nanomaterials on their toxicity.

Recent studies have shown that oxidative stress caused by nanomaterials results in excessive ROS production [9]. Nanomaterials can produce ROS by one-electron oxidative reactions with transition metal or nanomaterial surface groups [10, 11], or can directly impair mitochondria structure and function [12, 13]. The ROS induced by nanomaterials activates numerous signaling pathways, which may damage cell membranes, intracellular organelles, and nucleic acids, eventually leading to cell apoptosis or necrosis [9]. Interestingly, the body does not remain responsive to oxidative stress. For example, astrocytes produce functional extracellular mitochondria that support neuronal viability after stroke [14]. Furthermore, our previous study demonstrated that astrocyte-derived mitochondria can be transferred to neurons via tunneling nanotubes (TNTs) to fight CoNPs-induced neurotoxicity [15]. TNTs are characterized by their enrichment in F-actin (with few microtubes) and lack of attachment to the extracellular substrate [16]. TNTs can be transferred to many organelles, such as mitochondria [17], lysosomes [18], and even pathological proteins (tau [19], alpha-synuclein [20]). Among the substances transferred by TNTs, mitochondria are the most important organelle, as they can rescue energy production malfunction induced by toxicants [21]. However, whether this intercellular protection strategy via TNTs is common and universal among different engineered nanomaterials and the underlying mechanisms regulating TNTs formation remain unknown. A growing body of evidence has demonstrated that ROS is a major mechanism regulating TNTs formation [22]. As described above, ROS is the main product after nanomaterials exposure. However, the link between ROS production induced by engineered nanomaterials and TNTs formation has not yet been studied.

In this study, we aim to explore and compare whether different types of nanomaterials can induce TNTs formation (to the same degree), and investigate the potential role of ROS in TNTs formation and downstream molecular signaling pathways in response to various engineered nanomaterials. We hypothesized that engineered nanomaterials exposure induces cellular ROS and mitochondrial ROS production, which activates the downstream PI3K/AKT pathway, leading to the formation of TNTs. TNTs formation is an intercellular protective strategy that transfers by functional mitochondria to fight against nanomaterials-induced neurotoxicity. Thus, we investigated the toxic effects and TNTs formation of three types of engineered nanomaterials (CoNPs, TiO_2_NPs and MWCNTs) using mice primary astrocytes and human glioblastoma U251 cells. The properties of the three nanomaterials were fully characterized before the experiments. First, flow cytometry and high-content analysis were used to detect when and to what extent the nanomaterials entered the cells. The ability of the three nanomaterials to increase ROS/mtROS levels and induce cytotoxicity was examined. In addition, high-content dynamic observations and immunofluorescence were conducted to study the influence of nanomaterials on TNTs formation. What’s more, NAC (N-Acetylcysteine, a ROS scavenger) and MitoQ (Mitoquinone, an antioxidant targeting mtROS) were used to explore nanomaterials-induced TNTs formation. Finally, we explored the involvement of the PI3K/AKT/mTOR pathway in the nanomaterial-induced TNTs formation and mitochondrial transfer using various chemical inhibitors (Graphical abstract).

## Methods

### Characterization of TiO_2_NPs and MWCNTs

Cobalt nanoparticles (CoNPs, Cobalt–carbon-coated magnetic, nanopowder, ≥ 99%, Product number 697745, Batch Number MKCL5254), Titanium dioxide nanomaterials (TiO_2_NPs, anatase, nanopowder, ≥ 99.7%, Product Number 637254, Batch Number MKCK4358) and multi-walled carbon nanotubes (MWCNTs nanopowder, ≥ 98%, Product number 698849, Batch number MKBH5811V) were purchased from Sigma-Aldrich (USA). The nanomaterials were reconstituted with ddH_2_O and culture medium prior to characterization. The particle size was examined using Tecnai G2 F30 field emission transmission electron microscope (TEM) (FEI, USA) and quantified using Nano Measure 1.2. The dynamic light scattering (DLS), surface zeta potential measurements and Polydispersity were carried out on a Malvern Zetasizer Nano ZS instrument (Zetasizer Nano-ZS90, Malvern, UK).

### Preparation of Nanomaterials

To prepare stock solution, CoNPs, TiO_2_NPs and MWCNTs were diluted with ddH_2_O to a final concentration of 1 mg/mL in 1.5 mL microtubes, respectively. Before applying them to the cells, the solution was sonicated in a bath-type sonicator (KQ-500E, Kunshan Ultrasonic Instruments Co., LTD., China) for 10 min and shaken every three mins. Then, to reach the specific working concentration (such as 30 μg/mL), 60 μL solutions were added to 1940 μL 1640 medium without fetal bovine serum (FBS) (FCS500, Excell, Shanghai, China) to a final concentration of 30 μg/mL (working solution). The working solution was then used to culture cells. The same volume of water is added to the control group in all experiments as for solvent control.

### Cell culture and nanomaterial exposure

U251 human glioma cells were purchased from the State Key Laboratory of Genetic Resources and Evolution (Yunnan, China). U251 cells are a commonly used in vitro model to study neurotoxicity [23, 24], and are also widely utilized in studying TNTs formation and mitochondrial transfer [25–27]. Furthermore, we have also examined TNTs formation in the human neuroblast cells SH-SY5Y. Compared to SH-SY5Y cells, U251 cells exhibited a greater capability for TNTs formation under physiological conditions (Additional file 1: Fig. S1). Therefore, U251 cells were used to elucidate the mechanism underlying TNTs formation in depth.

U251 cells were cultured in 1640 medium (BL303A, Biosharp, Anhui, China) supplemented with 10% FBS and 100 units/mL of penicillin–streptomycin. Cells were cultured at 37 °C as monolayers in a humidified atmosphere containing 5% CO_2_.

When cell density reached 70–80% confluency, the medium was changed to 1640 without FBS. Cells were then treated with various concentrations of CoNPs, TiO_2_NPs and MWCNTs for 24 h for subsequent measurement.

To select the appropriate nanomaterials concentration, we measured the viability of U251 cells by exposing them to a series of nanomaterials concentrations. We selected the concentration with a similar degree of cell damage (30 μg/mL) across tested nanomaterials as the exposure concentration for the following study (Additional file 1: Fig. S2).

### The treatment of ROS scavengers and inhibitors

To scavenge ROS or mtROS, U251 cells were pretreated with 10 mM NAC (HY-B0215, MedChemExpress, New Jersey, USA) for 30 min or pretreated with 0.2 μM MitoQ (HY-100116A, MedChemExpress, New Jersey, USA) for 30 min and then to wait for measurement, prior to nanomaterials exposure. To inhibit the release of extracellular vesicles, U251 cells were pretreated with 10 μM GW4869 (HY-19363, MedChemExpress, New Jersey, USA) for 30 min prior to nanomaterials exposure. To inhibit TNTs formation, prior to nanomaterials exposure, 1 μM Latrunculin B (LAT-B) (HY-101848, MedChemExpress, New Jersey, USA) pretreated with cells for 30 min. To inhibit PI3K protein, U251 cells were pretreated with 10 μM LY294002 (HY-10108, MedChemExpress, New Jersey, USA) for 30 min prior to nanomaterials treatment. To inhibit AKT protein, U251 cells were pretreated with 10 μM Perifosine (HY-50909, MedChemExpress, New Jersey, USA) for 30 min prior to nanomaterials exposure. To inhibit mTOR, 25 nM Rapamycin (HY-10219, MedChemExpress, New Jersey, USA) was pretreated with U251 cells for 30 min prior to nanomaterials exposure.

### Primary astrocyte culture and exposure

Mice were housed in stainless steel cages in a ventilated animal facility at 22 ± 2 °C and relative humidity of 50 ± 10% under a 12 h light/dark cycle and fed with sterilized food and distilled water. All the mice were humanely treated throughout the experimental period.

Newborn C57BL/6 mice puppies (within 24 h) were euthanized by carbon dioxide inhalation. The cortex was dissected, and the meninges and blood vessels were removed in Hank's equilibrium salt solution (H1045, Solarbio, Beijing, China). Next, the minced cortex was transferred to F12 medium (BL305A, Anhui, China) containing 0.25% trypsin (25200056, Thermo Fisher Scientific, Massachusetts, USA) to digest at 37 °C for 30 min. After centrifugation and suspension, mixed glial cells were plated in a T-25 flask (156367, Thermo Fisher Scientific, Massachusetts, USA) coated with poly-lysine and cultured in DMEM medium (11965092, Thermo Fisher Scientific, Massachusetts, USA) containing 10% FBS. The cells were cultured at 37 °C in an atmosphere of 5% CO_2_ and 95% air. The cell culture medium was replaced every 24 h after plating and every two days. After 7–10 days, astrocytes were shaken at 250 RPM for 14 h at 37 °C to remove unwanted cells, including microglia, neurons, and fibroblasts. Astrocytes were digested with 0.25% trypsin at 37 °C for 5 min and seeded in 12-well plate for the following measurement.

Immunofluorescence was used to validate the purity of PA. Briefly, 4% w/v paraformaldehyde was added 12 well-plate and incubated at 4 °C for 15 min. The cells were permeabilized for 15 min with 0.15% Triton X-100 (ST795, Beyotime, Shanghai, China) in phosphate-buffered saline (PBS) (C0221A, Beyotime, Shanghai, China) and blocked with 10% normal goat serum (C0265, Beyotime, Shanghai, China) for 1 h at room temperature (RT). For GFAP staining, PA was incubated with anti-GFAP antibody (1:500) (Ab7260, Abcam, Cambridge, England) at 4 °C overnight. The Alexa-Fluora 488-conjugated secondary antibody was incubated at RT for 1 h, and 1 μg/mL DAPI (C1002, Beyotime, Shanghai, China) was used for nuclear staining. The purity of PA (%) = GFAP positive cells/DAPI positive cells × 100%. In total, 150 cells were counted in each well. The purity of PA was over 95% (Additional file 1: Fig. S3).

When PA density reached 70–80% confluence, the medium was changed to DMEM without FBS. Then, the PA was exposed to CoNPs, TiO_2_NPs and MWCNTs for 24 h before the next measurement.

**Fig. 1:**
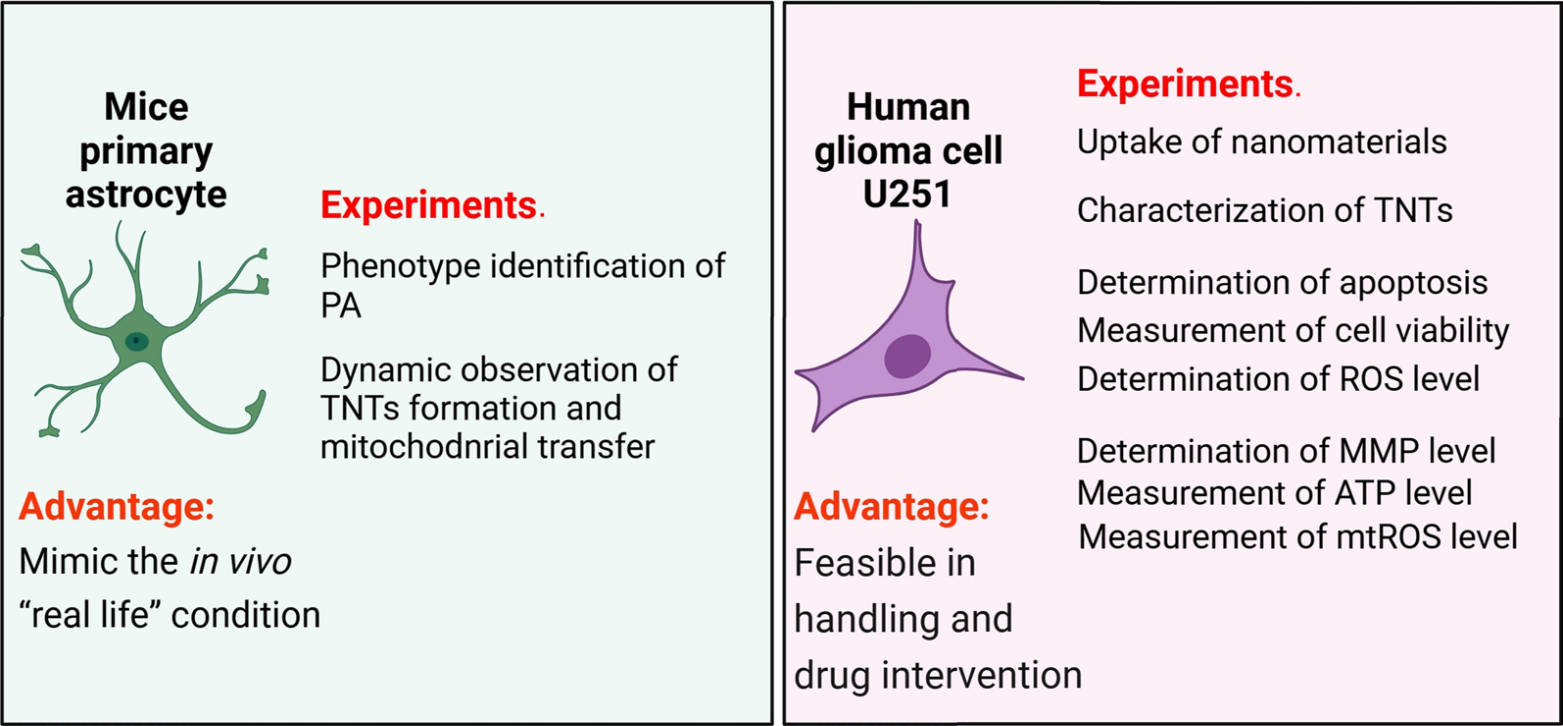
The two types of cells and their corresponding experiments were used in this study

The cell types used in each experiment are shown in Fig. 1.

### Cell viability assessment

U251 cells were seeded in a 96-well plate at a density of 5 × 10^3^ containing 100 μL cell medium and exposed to nanomaterials for 24 h. Then 10 μL CCK8 reagent (C0037, Beyotime, Shanghai, China) was added to wells and incubated at 37 °C for 1 h. A microplate reader (Multiskan FC, Thermo Fisher Scientific, Waltham, MA, USA) was used to measure the absorbance (A) at 450 nm. Six parallel wells were set up for each group, and the mean values were obtained. Cell survival rate was calculated using the formula: cell survival rate (%) = (Absorbance of the experimental group/ Absorbance of the control group) × 100%.

### High content screening system (HCS)

PA and U251 cells were seeded in the 24-well plates at a density of 2 × 10^3^ in each well. After nanomaterials exposure, the plate was observed using a high content screening system (PerkinElmer, Massachusetts, USA) for 24 h, and images were captured every 15 min.

### Quantification of TNTs and mitochondrial transfer

Using a TCS SP5 confocal microscope (Leica, Weitzlar, Germany), fields of sub-confluent cells were randomly selected with a 20X objective. At least ten images were obtained for each experimental group. The number of TNTs per one hundred cells was calculated for TNTs in U251 cells using Image J. At least fifty TNTs were imaged in each group. The percentage of TNTs containing mitochondria was quantified in each field.

### ATP measurement

U251 cells were seeded in 12-well plates at a density of 1 × 10^5^ for 24 h and transfected with the pCMV-Mito-AT1.03 plasmid (D2606, Beyotime, Shanghai, China) using lipo8000 (C0533, Beyotime, Shanghai, China) according to the manufacturer’s instruction. Afterwards, the transfected cells were exposed to nanomaterials. The images were captured using fluorescence microscope and the ATP intensity was quantified using Image J 2.1.

### Nanomaterials uptake

The uptake of nanomaterials was assessed using flow cytometry following methods reported by Suzuki et al. [28]. U251 cells treated with nanomaterials were washed three times with PBS to remove free particles. The cells were re-suspended in DMEM, and the number of particles taken up was analyzed by flow cytometry (FACSCanto II, Becton Dickinson, Franklin Lakes, USA). The sample profile was obtained by examining forward-scattered light (FSC) and side-scattered light (SSC). As each cell intercepts the path of the laser beam, the light that passes around the cell is measured as the FSC, indicating the cell size. The light scattered at a 90° angle to the axis of the laser beam was measured as the SSC and was related to intracellular density. Thus, the changes in cellular SS, after treatment with nanomaterials can be attributed to their uptake potential.

### Transmission electron microscope (TEM)

U251 cells were seeded in the 10 cm dish at a density of 1 × 10^6^, and then exposed to nanomaterials for 24 h. After being digested by trypsin, cells were centrifuged at 500 × *g* for 5 min into clumps. Subsequently, cells were fixed in 2.5% glutaraldehyde (P1126, Solarbio, Beijing, China) (diluted in 0.1 µM PBS; pH 7.4) at 4 °C for 24 h and then post-fixed in 1% osmium tetroxide (201030, Merck, New Jersey, USA) (dissolved in PBS; pH 7.4) at 25 °C for 60 min. After dehydration using different concentrations of Ethanol (30%, 50%, 80%, 90%, 100%), samples were embedded by resin (45347, Merck, New Jersey, USA) with different conditions (37 °C for 12 h; 45 °C for 12 h; 60 °C for 12 h) and ultrathin sectioning (the thickness is 50 nm), the samples were stained with uranyl acetate at RT for 60 min and stained with lead citrate (15326, Merck, New Jersey, USA) at RT for 8 min. Digital images were captured using TEM (FEI Tecnai G2 F30; Thermo Fisher Scientific, Inc.).

### Detection of mitochondrial reactive oxygen species (mtROS) and reactive oxygen species (ROS)

The mtROS and ROS levels in treated cells were measured using Mito-SOX (M36009, Invitrogen, Carlsbad, USA) and DCFH-DA dye staining (S0033S, Beyotime, Shanghai, China), respectively. Briefly, U251 cells were exposed to nanomaterials for 24 h, and then incubated with 0.5 μM Mito-Sox or 1 μM DCFH-DA for half an hour at 37 °C. Finally, the mtROS and ROS levels were measured using the fluorescence microscope (DMi8, Leica, Germany) at wavelengths of Ex/Em = 530 nm/562–588 nm and Ex/Em = 488 nm/515–545 nm, separately. To exclude the possible interference of nanomaterials’ autofluorescence on DCFH-DA and mitoSOX, we examined the emission and excitation wavelengths of the dyes and nanomaterials (methods and results in Additional file 1: Fig. S4A–D). In summary, nanomaterials’ autofluorescence would not interfere with the results of the DCFH-DA and mitoSOX probe.

### Measurement of mitochondrial membrane potential (MMP)

Cell MMP was detected by a JC-1 probe (C2005, Beyotime, Shanghai, China). Briefly, U251 cells were exposed to nanomaterials for 24 h, and then incubated with 1 μM JC-1 probe for 0.5 h at 37 °C. Finally, MMP was measured by fluorescence microscopy, and quantified by Image J according to literature [29].

### Western blot

Exposed U251 cells were washed three times with cold PBS, collected, and lysed with 120 μL ice-cold RIPA lysis buffer (P0013D, Beyotime, Shanghai, China). Afterwards, cell-free supernatants were obtained by centrifugation of the lysates at 12 000 × *g* for 25 min at 4 °C. Sodium dodecyl sulfate (SDS) loading buffer was added to each supernatant, and boiled for 10 min to generate SDS-PAGE samples. The 15 μg samples were electrophoresed on a 10% SDS polyacrylamide gel. Proteins were transferred onto a polyvinylidene fluoride membrane. After blocking the membrane with 5% nonfat milk in Tris-buffered saline containing 0.1% Tween-20 (TBST) (ST671, Beyotime, Shanghai, China) for 1 h at 25 °C, the blots were incubated with primary antibodies of interest overnight at 4 °C. After washing with TBST five times, the blots were incubated with a peroxidase-conjugated secondary antibody. Antibodies binding was detected by chemiluminescent staining using an ECL detection kit (RPN2235, Amersham, USA). The grayscale of the protein bands was analyzed using Image J software. Primary antibodies were used at the following concentrations: p-mTOR (1:2000, AF5869, Beyotime, China), mTOR (1:2000, AF1648, Beyotime, China), P110 (1:2000, AF1966, Beyotime, China), P85 (1:2000, AF7742, Beyotime, China), p-PI3K (1:2000, AF5905, Beyotime, China), AKT (1:2000, AA326, Beyotime, China), p-AKT (1:2000, AF1546, Beyotime, China), beta-ACTIN (1:3000, 81115-1-RR, Proteintech, China), anti-rabbit peroxidase-conjugated secondary antibodies (1:10,000, A16110, ThermoFisher, USA).

### Statistical analysis

Data were analyzed using SPSS software (version 19.0, IBM Corporation, Armonk, NY, USA). A one-way analysis of variance (ANOVA) was used for multiple comparisons. Experimental data with heterogeneous variance were analyzed using the Kruskal–Wallis nonparametric test for different exposure groups. A *P* value < 0.05 indicates statistical significance. All experiments were carried out in independent triplicates and three individual experiments were performed unless otherwise specified.

## Result

### Characterization of nanomaterials

**Fig. 2:**
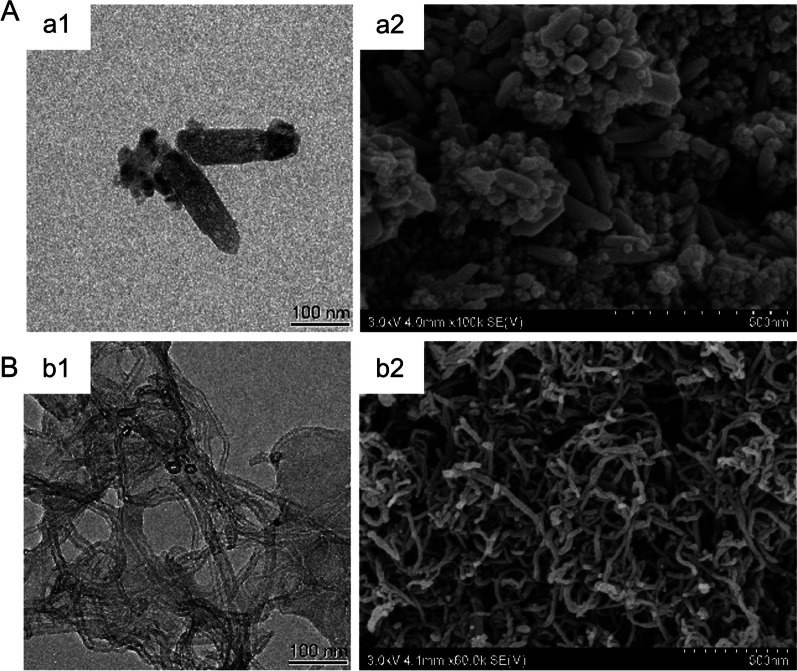
Images of TiO_2_NPs and MWCNTs captured by TEM and SEM. (a1, b1) TEM images show the size of 1 mg/mL TiO_2_NPs and MWCNTs in water. Scale bar = 100 nm. (a2, b2) SEM images demonstrate the morphology of 1 mg/mL TiO_2_NPs and MWCNTs in water. Scale bar = 500 nm

First, the properties of three nanomaterials were characterized. The physical properties of CoNPs have been demonstrated in our previously published literature [15]. The purity of TiO_2_NPs and MWCNTs was over 98%, and the endotoxin level was below the detection limit (0.01 EU/mL) at the concentration of 1 mg/mL, much higher than the concentration administrated (Additional file 1: Fig. S5A, B). As shown in the TEM and SEM results (Fig. 2), TiO_2_NPs was generally a long cylinder with a diameter of 36.43 nm (16.44–52.33 nm), while MWCNTs was a long, tubular structure with a diameter of 22.12 nm (10.14–36.97 nm). The Z-average, polydispersity and zeta potential of nanomaterials in both water and medium were demonstrated in Table 1. In brief, there was little change in water and medium for all nanomaterials in polydispersity.

**Table 1:** Characteristics of nanomaterials

Characteristic	TiO_2_NPs	MWCNTs	Technique
Source	Sigma, USA	Sigma, USA	
Purity	≥ 99.7%	≥ 98%	Trace metal analysis
Endotoxin	Under detection limit (0.01 EU/mL)	Under detection limit (0.01 EU/mL)	TAL
Size (nm)	36.43 (16.44–52.33)	22.12 (10.14–36.97)	TEM
Z-average (d.nm)	In water: 640 In medium: 687.2	In water: 581.4 In medium: 1559	DLS
Polydispersity	In water: 0.858 In medium: 0.871	In water: 0.467 In medium:0.449	
Zeta potential (mV)	In water: − 4.82 In medium: − 11.5	In water: 4.45 In medium: − 26.9	

DLS: Dynamic light scattering; TAL: Tachypleus Amebocyte Lysate (1 mg/mL suspension was used). Z-average, Polydispersity and Zeta potential were tested in water and 1640 medium

### Nanomaterials promote TNTs formation and mitochondrial transfer

**Fig. 3:**
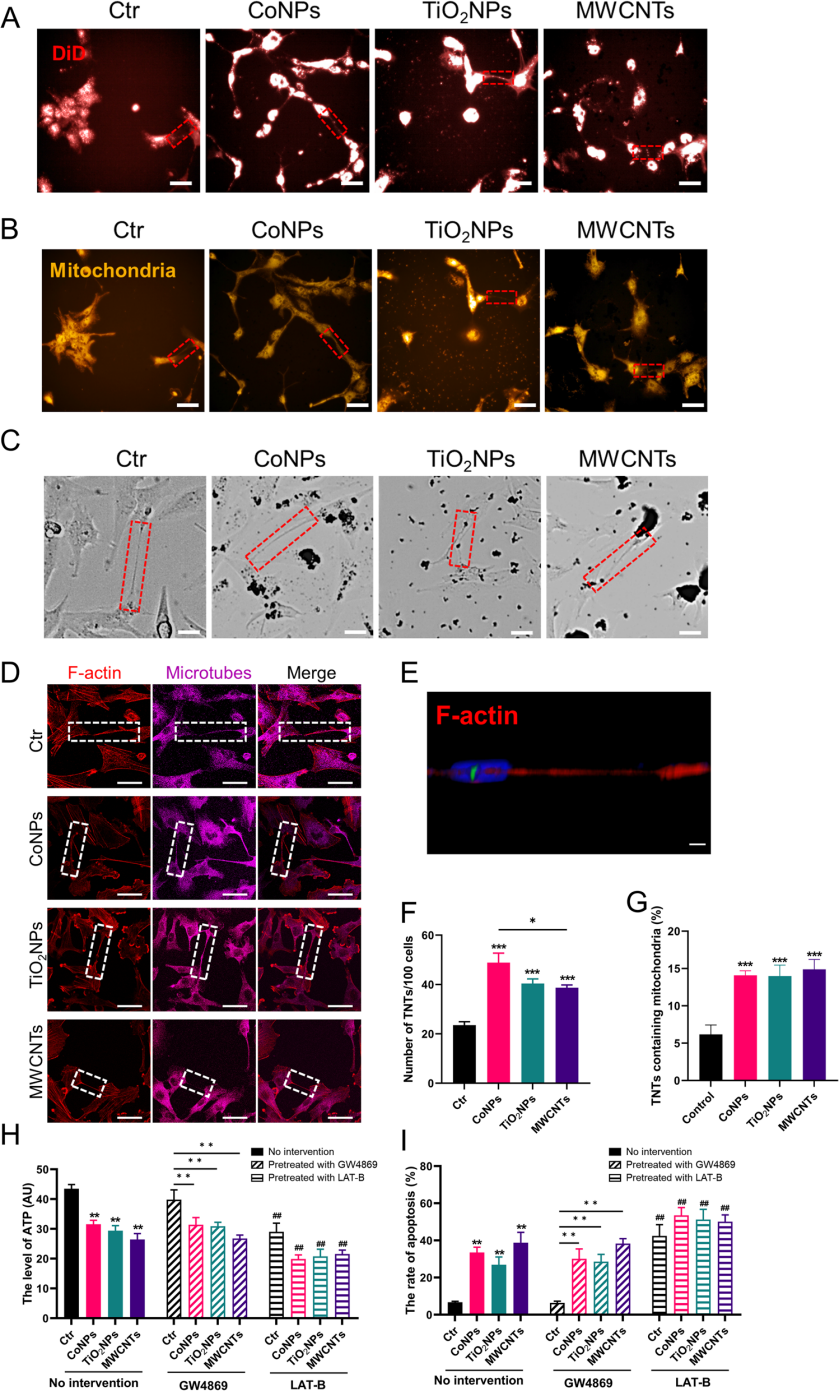
Nanomaterials induce TNTs formation and mitochondrial transfer. **A** Representative image of TNTs after exposure to nanomaterials in primary astrocyte. **B** Representative image of mitochondrial transfer via TNTs under nanomaterials treatment in primary astrocyte. **C** Representative bright field images of TNTs like in U251 cells. **D** Representative fluorescent images of TNTs in U251 cells after exposure to nanomaterials shown the TNTs structure. **E** 3D reconstruction of TNTs in U251 cells used ImageJ. **F** Quantification analysis of TNTs in U251 cells. **G** Quantification analysis of mitochondrial transfer via TNTs in U251 cells. 10 μM GW4869 or 1 μM LAT-B were pretreated with cells for 30 min prior to nanomaterial exposure. **H** The level of ATP in U251 cells after exposure to nanomaterials. **I** The ratio of apoptosis in U251 cells upon nanomaterials treatment. Scale bar = 25 μm. *, ** and *** indicate *P* < 0.05, 0.01 and 0.001 compared with the control group. ^**##**^ indicates *P* < 0.01 compared with the respective nanomaterials group (regarding the no intervention nanomaterials group). Date present as mean ± SEM. n = 3

First, we used mouse primary cortical astrocytes (PA) to examine TNTs formation and mitochondrial transfer after nanomaterial exposure. We recently reported that in response to CoNPs exposure, astrocytes transfer functional mitochondria to damaged neurons via TNTs [15]. Thus, in combination with DiD, a cell membrane dye probe was used to label TNTs, and MitoTraker Red was used to label mitochondria in this study. A high content screening system (HCS) was used to dynamically observe TNTs formation and mitochondrial transfer continuously for 24 h, and images were captured every 15 min. As observed in HCS, PA is in close contact and undergoes membrane fusion, and PA migrates away from each other, drawing out membrane tethers and leading to the formation of TNTs. This process is recognized as “cell dislodgment” [30]. Simultaneously, vesicles transfer occurred actively in TNTs (Fig. 3A). The speed of vesicles in the control group is 0.81 μm/min (Additional file 2: Video S1), in the CoNPs group is 0.51 μm/min (Additional file 3: Video S2), in the TiO_2_NPs group is 0.34 μm/min (Additional file 4: Video S3), and in the MWCNT group is 0.56 μm/min (Additional file 5: Video S4). Mitochondrial transfer via TNTs also observed (Fig. 3B). The rate of mitochondrial transfer in the control group was 0.92 μm/min (Additional file 6: Video S5). In contrast, in response to CoNPs exposure, mitochondrial transfer is slower, i.e., 0.50 μm/min (Additional file 7: Video S6), 0.45 μm/min (Additional file 8: Video S7) and 0.57 μm/min (Additional file 9: Video S8) in TiO_2_NPs and MWCNTs treated groups, respectively.

Afterwards, the human glioblastoma cell line U251 was selected to investigate the mechanism of TNTs formation because it is a widely used glial cell model to study neurotoxicity [23, 24] and TNTs formation in the central nervous system (CNS) [25, 26]. U251 cells were exposed to the nanomaterials for 24 h, followed by HCS examination to elucidate the mechanism of TNTs formation. Thin membranous bridges connecting the two cells were observed in the bright field image, suggesting the formation of a TNTs-like structure in U251 cells (Fig. 3C). One of the most important characteristics of TNTs is the enrichment of F-actin (either with or without microtubes) and non-attachment to extracellular substrates [16]. As demonstrated, the TNTs induced by nanomaterials between U251 cells consisted of F-actin and microtubules (Fig. 3D). Furthermore, as demonstrated by 3D reconstruction, the observed TNTs were not attached to the extracellular substrate (Fig. 3E). In addition, the data indicated that U251 cells could act as a model to study TNTs formation in CNS.

Finally, we investigated whether there was a difference in the number of TNTs stimulated by the three types of nanomaterials. Quantitative analyses revealed that the percentage of TNTs significantly increased upon nanomaterial exposure in U251 cells (Fig. 3F). The number of TNTs stimulated by CoNPs appeared to be the highest, followed by TiO_2_NPs and MWCNTs. In addition, the mitochondrial transfer via TNTs also increased, suggesting a protective role of TNTs formation in U251 cells (Fig. 3G). All nanomaterials induced mitochondrial transfer, consistent with increased TNTs. Finally, to investigate the function of TNTs and mitochondrial transfer upon nanomaterials exposure, LAT-B, a specific TNTs inhibitor, and GW4869, an extracellular vesicle inhibitor, were utilized in the co-culture system. Nanomaterials exposure reduced the ATP level in U251 cells. GW4869 did not influence ATP levels, whereas LAT-B exacerbated ATP reduction induced by nanomaterials (Fig. 3H, Additional file 1: Fig. S6A). Simultaneously, the apoptosis of U251 cells was also examined, and it was found that LAT-B further aggravated the apoptosis of cells induced by nanomaterials, whereas GW4869 did not affect cell apoptosis upon nanomaterials exposure (Fig. 3I, Additional file 1: Fig. S6B).

All the above results confirm that nanomaterials induce TNTs formation and mitochondrial transfer via TNTs but not extracellular vesicles (EVs), in both PA and U251 cells. In contrast, the number of TNTs induced by different nanomaterials is different. However, the potential mechanism(s) of the nanomaterials-induced TNTs formation requires further investigation.

### Nanomaterials enter U251 cells and induce neurotoxicity

A growing body of evidence demonstrates that TNTs is associated with environmental stressors, such as ischemia, stroke, and hypoxia. Impaired cells actively extend protrusions towards “healthy” cells to form TNTs. Therefore, we propose that the differences in TNTs numbers induced by nanomaterials were due to the different degrees of damage caused by the nanomaterials.

**Fig. 4:**
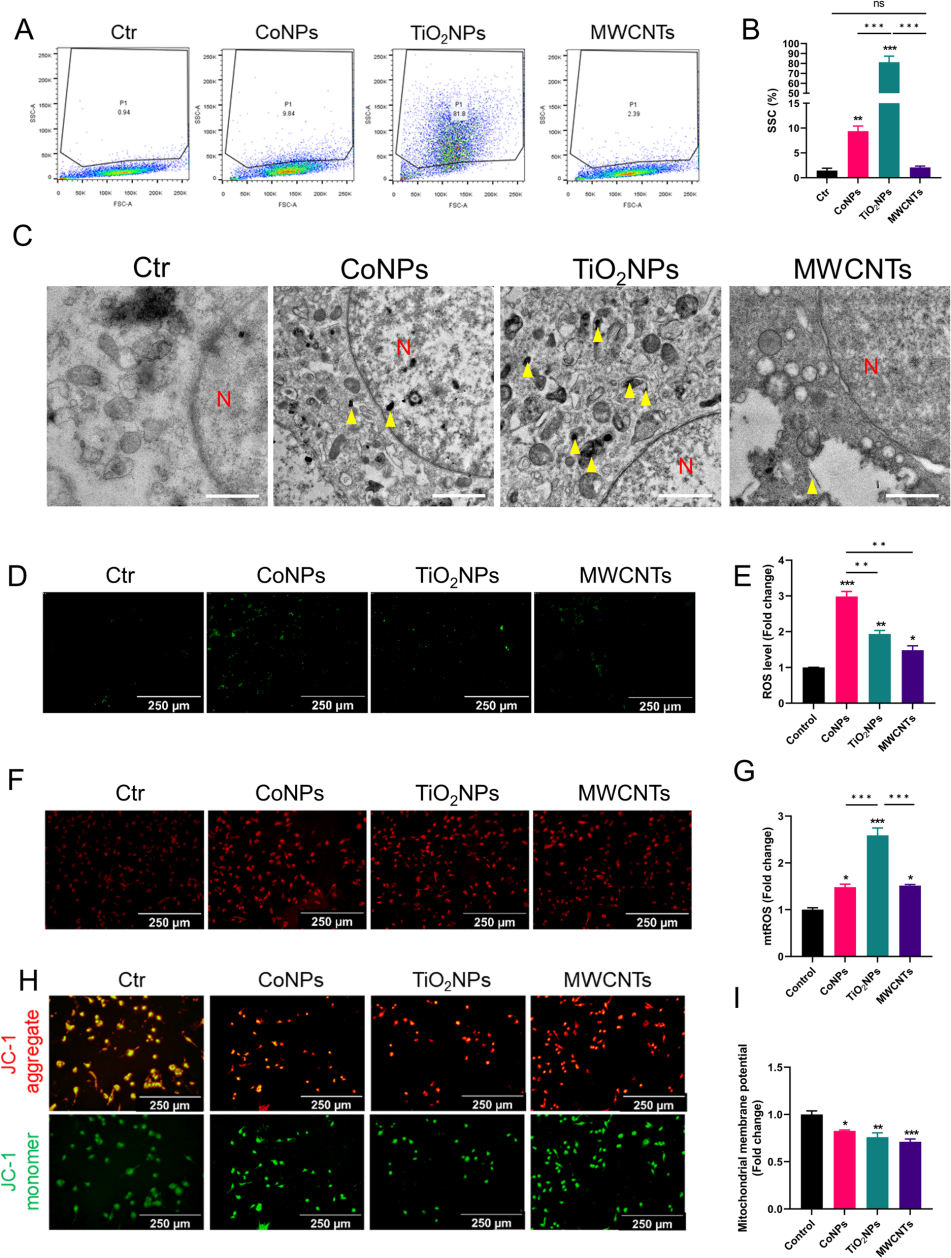
Nanomaterials induce ROS/mtROS generation and mitochondrial damage. **A** U251 cells absorbed the nanomaterials measured by flow cytometer. **B** Statistics of absorption ratio characterized by SSC in U251 cells. **C** Nanomaterials were absorbed by U251 cells detected by TEM. Scale bar = 500 nm. **D** Nanomaterials induced the ROS generation detected by fluorescence microscope in U251 cells. **E** Quantitative analysis of ROS level in U251 cells. **F** Nanomaterials induced the production of mtROS detected by fluorescence microscope in U251 cells. **G** Quantitative analysis of mtROS level in U251 cells. **H** Nanomaterials impaired MMP levels measured by fluorescence microscope in U251 cells. **I** Quantitative analysis of MMP levels in U251 cells. Scale bar = 250 μm. *, ** and *** indicate *P* < 0.05, 0.01 and 0.001 compared with control group. N: Nucleus. The yellow arrow represents nanomaterials absorbed by U251 cells. Date present as mean ± SEM. n = 3

First, the uptake of nanomaterials by U251 cells was examined by flow cytometry. Corroborating the dispersion and polydispersity of three types of nanomaterials (Fig. 3C and Table 1), TiO_2_NPs was the most absorbed by cells, and the SSC pattern became significantly discrete (90% of SSC). CoNPs, with only 0.751 dispersion in SSC, was the next, followed by MWCNTs, with almost no change in SSC (Fig. 4A, B). In addition, TEM was utilized to further verify the uptake of nanomaterials by U251 cells. In line with the flow cytometry results, all nanomaterials could enter U251 cells. CoNPs were more gathered to nucleus, with some entering nucleus. On the contrary, TiO_2_NPs mostly surrounded with membrane structure around nucleus, while MWCNTs with cavity structure (Fig. 4C). Consistent with flow cytometry, HCS also demonstrated that U251 cells began taking up nanomaterials at 1.5 h and reached the uptake peak at 6 h (Additional file 10: Video S9). Next, ROS levels in U251 cells were measured using DCFH-DA. All nanomaterials promoted ROS generation compared with the control group. Although most TiO_2_NPs was absorbed by U251 cells, ROS production was only secondary to CoNPs-induced ROS production. The ROS levels induced by MWCNTs is the lowest (Fig. 4D, E). At the same time, mtROS was detected via mitoSOX probe in U251 cells. In contrast to the above results for ROS, mtROS induced by TiO_2_NPs was the highest, followed by CoNPs and MWCNTs (Fig. 4F, G). Finally, the functional status of mitochondria in U251 cells was examined by mitochondrial membrane potential (MMP) via JC-1 probe. MWCNTs significantly decreased the MMP of U251 cells, followed by the reduction induced by TiO_2_NPs. CoNPs caused the least MMP reduction, which was still higher than that in the control group, indicating the damage to mitochondria (Fig. 4H, I). In summary, different types of nanomaterials cause varying degrees and types of damage. CoNPs caused a significant increase in ROS levels, TiO_2_NPs mainly increased mtROS levels, and the toxicity induced by MWCNTs was the lowest, consistent with the lowest amount of cellular uptake.

### ROS/mtROS increases TNTs formation and mitochondrial transfer after nanomaterials exposure

**Fig. 5:**
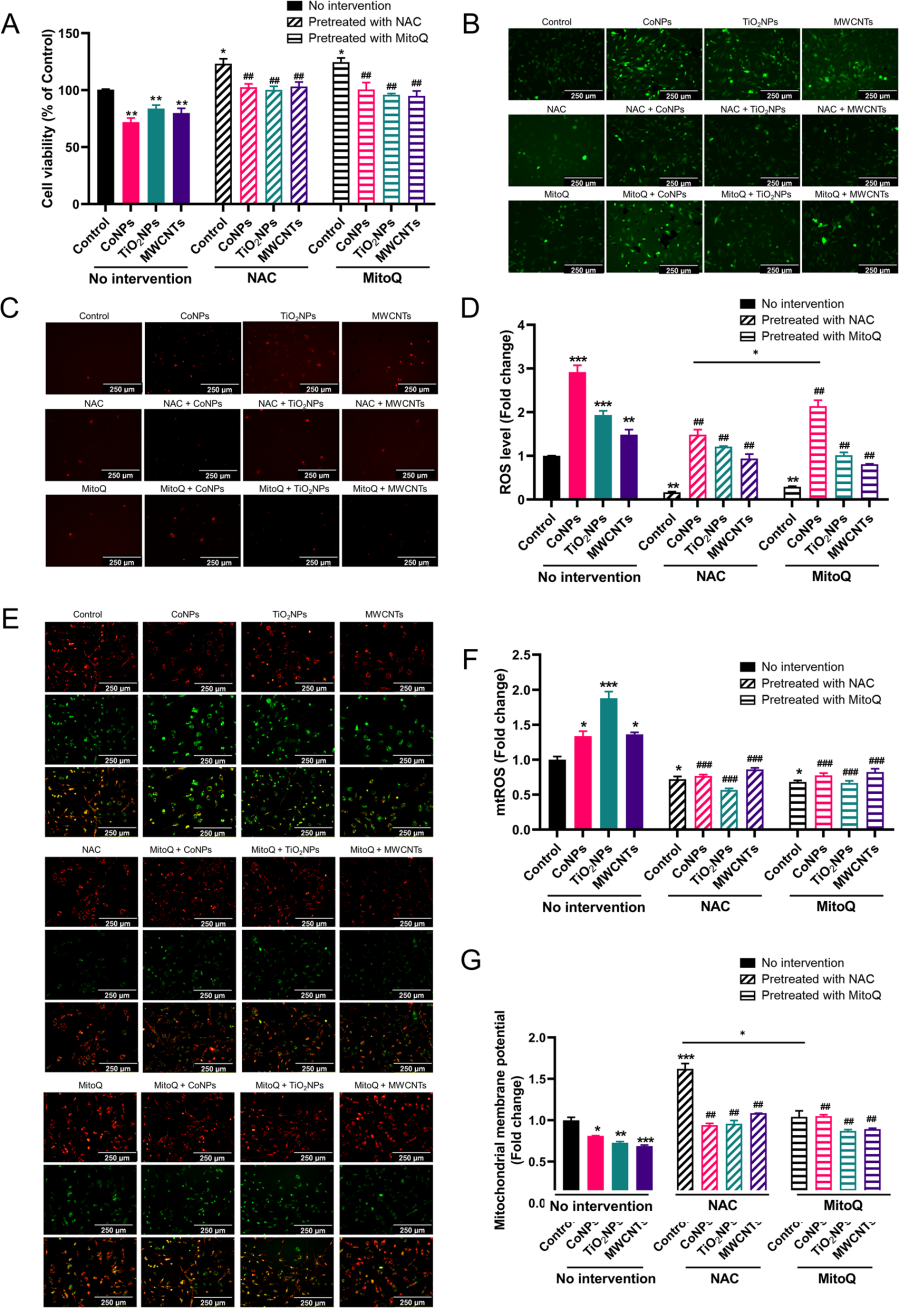
NAC/MitoQ decreases ROS/mtROS levels and relieves mitochondrial damage induced by Nanomaterials in U251 cells. **A** U251 cells were pretreated with 10 mM NAC for 30 min or pretreated with 0.2 μM MitoQ for 30 min and then were measured by CCK8 after being exposed to nanomaterials for 24 h. **B**, **D** Representative image of ROS level and quantification of ROS level after exposure to nanomaterials in U251 cells. **C**, **F** Representative image of mtROS level and its corresponding quantification analysis in U251 cells. **E**, **G** Representative image of MMP measure by JC-1 and its quantification analysis in U251 cells. Scale bar = 250 μm. *, ** and *** indicate *P* < 0.05, 0.01 and 0.001 compared with control group. ^**##**^ and ^**###**^ indicate *P* < 0.01 and 0.001 compared with the respective nanomaterials group (regarding the no intervention nanomaterials group). Date present as mean ± SEM. n = 3

We then investigated whether the generation of ROS/mtROS is key to nanomaterial-induced TNTs formation. Because the ROS/mtROS are the major mechanism for nanotoxicity, NAC, a ROS scavenger [31], and MitoQ, a mitochondrial antioxidant [32], were used to pretreat U251 cells before nanomaterial exposure. NAC and MitoQ rescued the nanomaterial-induced decrease in U251 cell viability (Fig. 5A). Simultaneously, both NAC and MitoQ reduced the level of ROS (Fig. 5B, D) and mtROS (Fig. 5C, F) induced by nanomaterials in U251 cells. To exclude potential false positive results of ROS, the ROS positive controls, Rosup, were used in U251 cells. Rosup significantly increased ROS levels compared with nanomaterials exposure, which abolished by NAC and mitoQ pretreatment, indicating that NAC and MitoQ could indeed reduce ROS levels (Additional file 1: Fig. S7A, B). In addition, the reduction in MMP in U251 cells was reversed by both NAC and MitoQ pretreatment (Fig. 5F, G). In brief, we demonstrated that reducing ROS/mtROS reversed nanomaterials-induced cellular and mitochondrial toxicity.

**Fig. 6:**
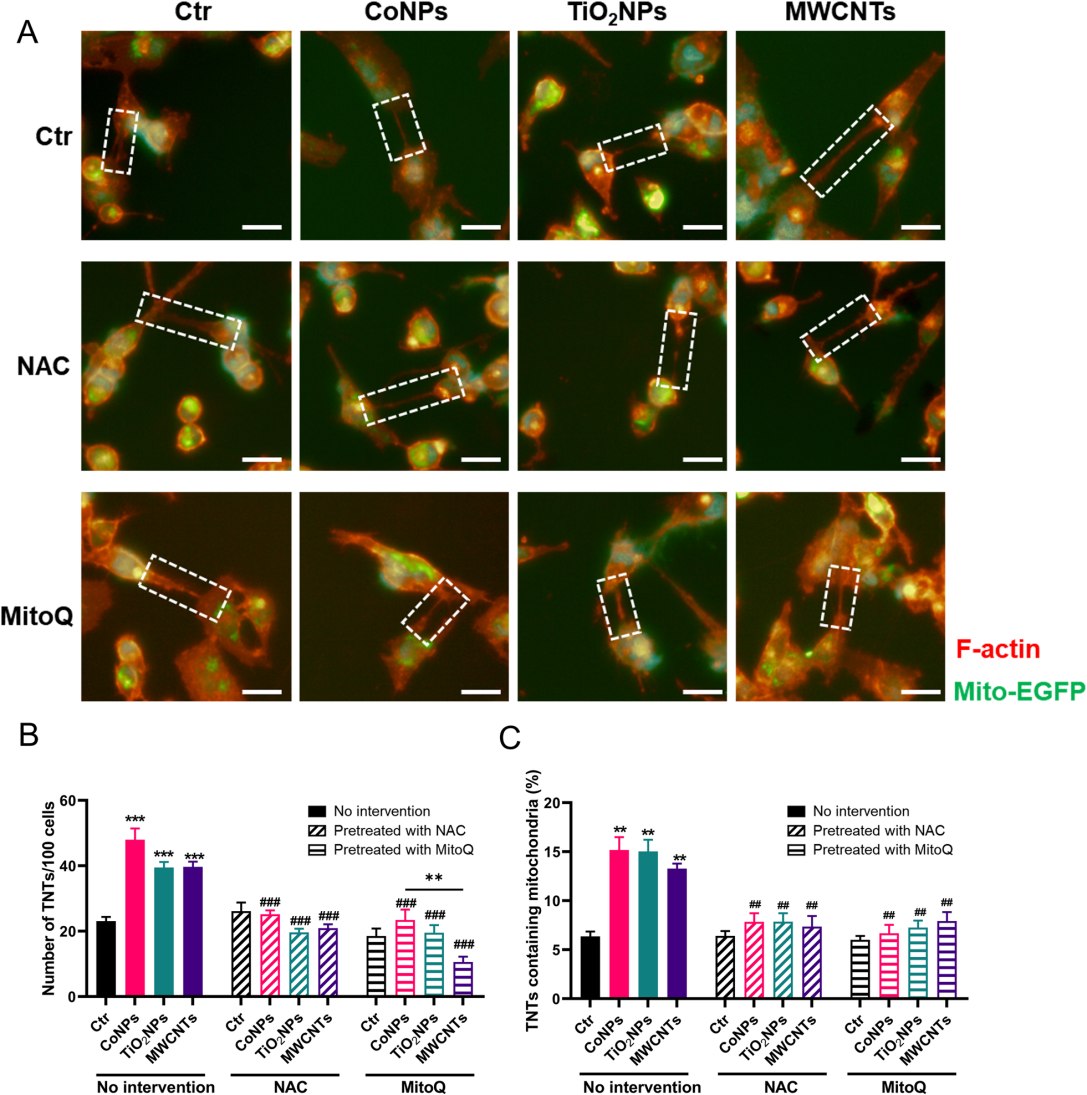
NAC/MitoQ decreases the TNTs formation and mitochondrial transfer induced by nanomaterials. U251 cells were pretreated with 10 mM NAC or 0.2 μM MitoQ for 30 min and then exposed to nanomaterials. **A** Representative image of TNTs in U251 cells after exposure to nanomaterials. **B** Statistic analysis of TNTs numbers in U251 cells. **C** Quantification analysis of mitochondrial transfer in U251 cells. Scale bar = 250 μm. ** and *** indicate *P* < 0.01 and 0.001 compared with the control group, ^**##**^ and ^**###**^ indicate *P* < 0.01 and 0.001 compared with the respective nanomaterials group (regarding the no intervention nanomaterials group). Scale bar = 25 μm. Date present as mean ± SEM. n = 3

Then we examined the relationships between ROS/mtROS, and TNTs formation and mitochondrial transfer. Interestingly, NAC was more capable of eliminating ROS/mtROS than MitoQ in U251 cells. Nevertheless, the abilities of NAC and MitoQ to reduce TNTs number were similar (Fig. 6A, B). What’s more, mitochondrial transfer was also reduced after NAC and MitoQ pretreatment of U251 cells (Fig. 6C). Combined with the results shown in Fig. 4, these results indicate that TNTs formation has a strong relationship with ROS/mtROS levels, which was not closely related to mitochondrial damage. CoNPs induced the highest levels of ROS, and TiO_2_NPs induced the highest levels of mtROS in U251 cells. Although MWCNTs induced the largest decrease in MMP, the TNTs induced by MWCNTs was the lowest (while still significantly higher than that of the control group). In summary, ROS/mtROS induced by nanomaterials is the major mechanism that induces TNTs development, which can be abolished by the ROS scavengers NAC and MitoQ.

### ROS/mtROS regulates TNTs formation via the PI3K/AKT/mTOR pathway following nanomaterials exposure

**Fig. 7:**
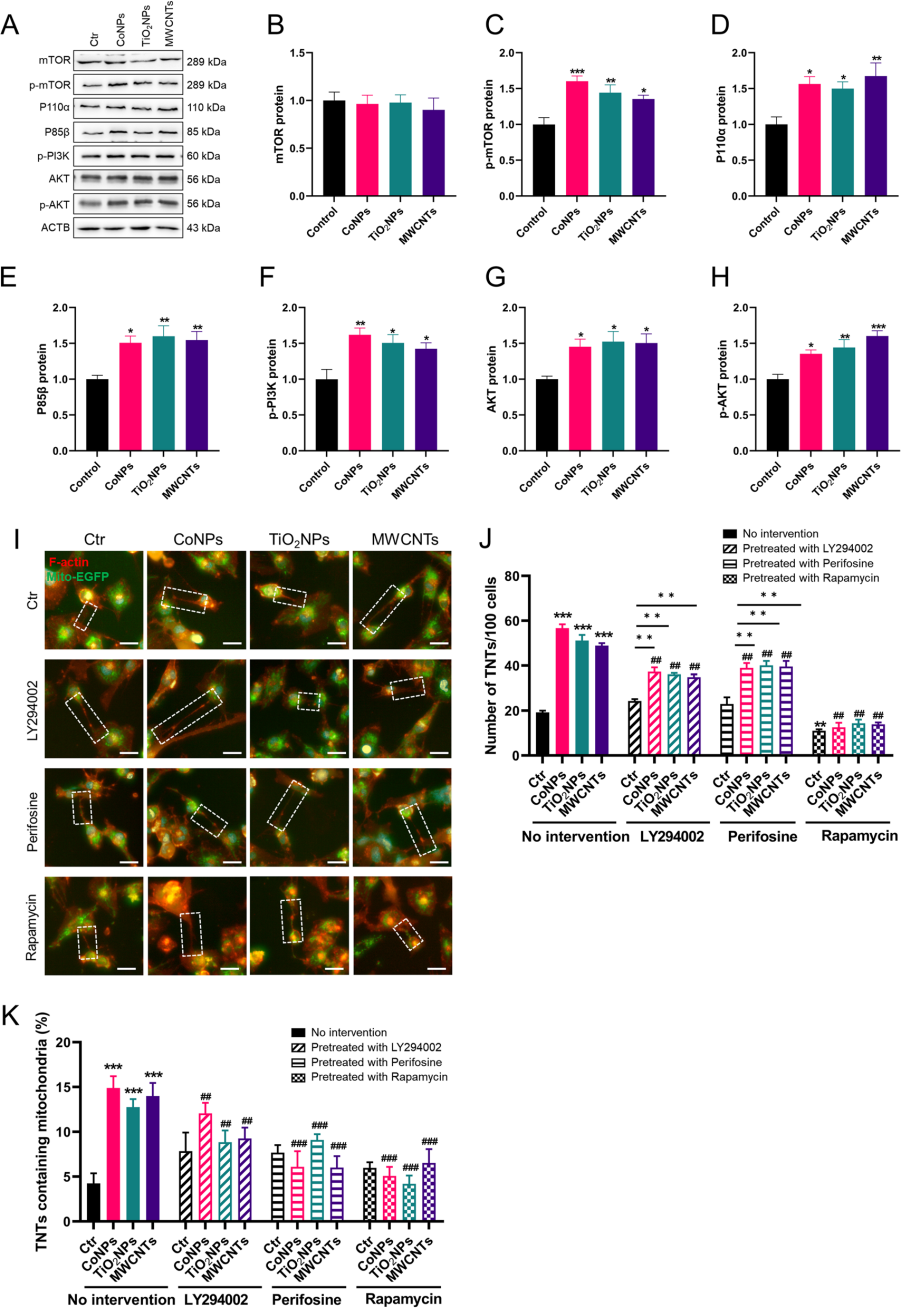
PI3K/AKT/mTOR pathway participates in the TNTs formation upon nanomaterials exposure in U251 cells. Cells were pretreated with 10 μM LY294002, 10 μM Perifosine or 25 nM Rapamycin for 30 min and then exposed to nanomaterials. **A** Western blot analysis of the PI3K PI3K/AKT/mTOR pathway in U251 cells. **B**–**H** Statistic analysis of PI3K/AKT/mTOR pathway in U251 cells. **I** Representative fluorescent image of TNTs in U251 cells. **J** Quantification of TNTs number in U251 cells. **K** Quantification of mitochondrial transfer via TNTs in U251 cells. Scale bar = 25 μm. *, ** and *** indicate *P* < 0.05, 0.01 and 0.01 compared with control group. ^**##**^ and ^**###**^ indicate *P* < 0.01 and 0.001 the respective nanomaterials group (regarding the no intervention nanomaterials group). Scale bar = 25 μm. Date present as mean ± SEM. n = 3

ROS/mtROS regulated TNTs formation, as demonstrated above (Fig. 6); however, the specific mechanism remains obscure. The PI3K/AKT pathway is critical in metabolism, proliferation, cell survival, and angiogenesis in response to extracellular signals, including nanomaterials-induced cytotoxicity [33]. At the same time, the PI3K/AKT pathway participates in TNTs formation upon H_2_O_2_ exposure [34]. PI3K promotes the re-localization of AKT to the plasma membrane, which is phosphorylated for full activation [35], which may act on TNTs formation. However, whether this pathway mediates the nanomaterial-induced TNTs formation remains unclear. Therefore, we first found that three nanomaterial types activated the expression of P110α and P85β (the two PI3K isoforms) in U251 cells. At the same time, the three nanomaterials activated AKT and its phosphorylated isoforms in U251 cells. These results indicated that the PI3K/AKT pathway was activated under nanomaterial exposure. Because mTOR is a common downstream effector of the PI3K/AKT pathway, total and activated p-mTOR expression was checked in U251 cells after nanomaterials exposure. While the total mTOR protein did not change, p-mTOR increased after exposure, indicating that p-mTOR was activated by three types of nanomaterials in U251 cells (Fig. 7A–H).

However, the causal relationship between PI3K/AKT/mTOR activation and TNTs formation in response to nanomaterials exposure requires further investigation. Hence, the PI3K inhibitor LY294002, the AKT inhibitor Perifosine and mTOR inhibitor Rapamycin were utilized. In U251 cells, all the inhibitors significantly reduced the numbers of TNTs induced by nanomaterials, while Rapamycin was the more potent inhibitor of TNTs. Consistently, the number of transferred mitochondria in U251 cells decreased in the presence of inhibitors (Fig. 7I–K). These results indicated that the PI3K/AKT/mTOR pathway participates in nanomaterials-induced TNTs formation.

**Fig. 8:**
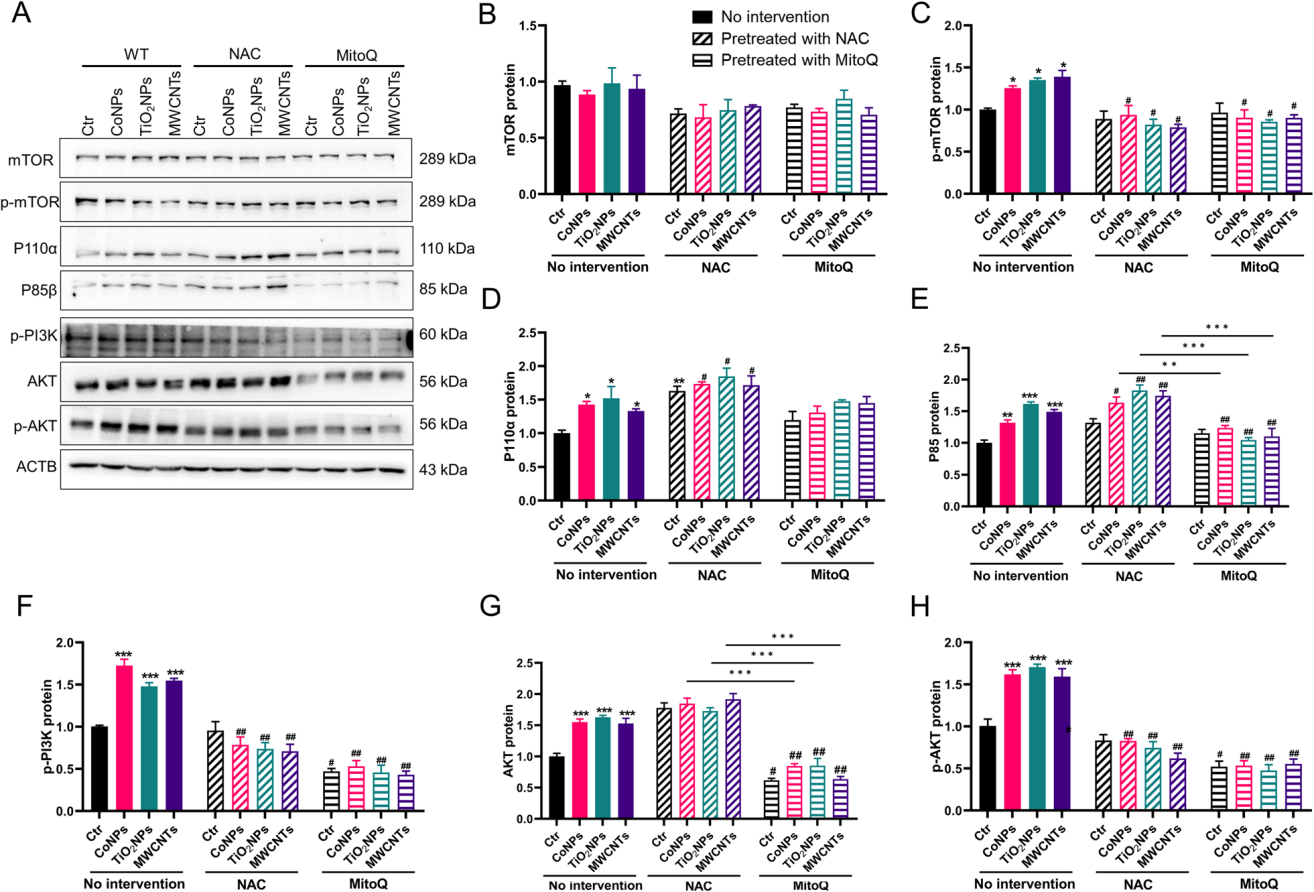
ROS/mtROS regulates TNTs formation under nanomaterials exposure via PI3K/AKT/mTOR pathway in U251 cells. Cells were pretreated with 10 mM NAC or 0.2 μM MitoQ for 30 min and then exposed to nanomaterials for 24 h. **A** Western blot analysis of the PI3K/AKT/mTOR pathway in U251 cells. **B**–**H** Statistic analysis of PI3K/AKT/mTOR pathway in U251 cells. *, ** and *** indicate *P* < 0.05, 0.01 and 0.01 compared with control group. ^**#**^ and ^**##**^ indicate *P* < 0.05 and 0.01 compared with the respective nanomaterials group (regarding the no intervention nanomaterials group). Date present as mean ± SEM. n = 3

As previously demonstrated, the three nanomaterials induced ROS/mtROS production and, stimulated TNTs formation and mitochondrial transfer. We further investigated whether ROS/mtROS-induced TNTs formation depends on the PI3K/AKT/mTOR pathway. Therefore, we examined alterations in the PI3K/AKT/mTOR pathway after applying the ROS scavenger NAC and the mtROS scavenger MitoQ in U251 cells. Interestingly, NAC increased the expression of P110α, and P85β, while the phosphorylation of PI3K was decreased after NAC pretreatment, and as for MitoQ, P110α P85β and p-PI3K expression decreased under nanomaterial exposure. Most importantly, the total AKT protein was reduced under MitoQ pretreatment but not under NAC. Phosphorylated AKT levels decreased after NAC and MitoQ treatment. Furthermore, NAC and MitoQ inhibited mTOR phosphorylation (Fig. 8A–H). In summary, ROS/mtROS promotes TNTs formation stimulated by nanomaterials via activating the PI3K/AKT/mTOR pathway.

## Discussion

An increasing number of engineered nanomaterials have been manufactured and utilized in the environment, making their toxicity a public health concern. CoNPs, TiO_2_NPs and MWCNTs are widely accepted as engineered nanomaterials that can enter the body and reach the central nervous system, raising concerns about their neurotoxicity. The original discovery of TNTs was closely related to homeostasis and pathogenesis [36, 37], especially in neurotoxicity induced by oxidative stress [38]. Here, we show, for the first time, that three types of engineered nanomaterials can promote TNTs formation and mitochondrial transfer via the induction of oxidative stress, a common protective strategy in response to nanomaterial exposure that restores ATP production and cell viability. Most importantly, the sophisticated mechanism of TNTs formation was fully elucidated.

Our group recently reported the transfer of mitochondria via TNTs against CoNPs-induced neurotoxicity [15]. However, further investigations are required to determine whether TNTs formation and mitochondrial transfer are universal in response to other nanomaterials. Here, we present evidence that three engineered nanomaterials exposure can induce TNTs formation in primary astrocytes and U251 cells. Moreover, the number of TNTs formed in the cells significantly increased upon nanomaterials exposure. Surprisingly, the model of TNTs formation is consistent with ‘cell dislodgment’, in which cells are in close contact and membrane fusion, and cells migrate away from each other, drawing out membrane tethers, leading to the formation of TNTs [30].

A growing body of evidence has shown that TNTs can protect cells from environmental stress due to their capacity to transfer materials between cells, such as mitochondria [17] and lysosomes [18]. For example, healthy N2a cells can donate their mitochondria to exposed-H_2_O_2_ N2a or ρ^0^ N2a (mitochondrial-DNA depleted cells) to improve apoptotic, oxidative stress, autophagic, and mitochondrial or DNA-damaged biomarkers indices. Consistent with this finding, we observed mitochondrial transfer in primary astrocytes and U251 cells after nanomaterials exposure. More importantly, the number of mitochondria transferred via TNTs increased after nanomaterials exposure. TNTs and mitochondrial transfer significantly protected neural cells from ATP reduction and cell apoptosis induced by nanomaterials. Interestingly, vesicles pre-labeled with DID were exchanged between primary astrocytes. However, the substances contained in the vesicles are unclear and warrant further investigation. It has been reported that vesicles can carry many substances, such as proteins, mRNA, and mitochondria. One study indicated that protein-containing vesicles can be transferred via TNTs to function biological process [22]. Compared with EVs, vesicle transfer via TNTs is faster and more accurate [39].

Although the three types of engineered nanomaterials have different biophysical properties (size, Z-average, polydispersity and zeta potential), they share the same trend of TNTs formation and mitochondrial transfer in response to nanomaterials exposure. The results indicated that a common mechanism regulates TNTs formation and mitochondrial transfer, regardless of the nanomaterial properties. ROS is a major regulator of TNTs formation [22]. After cellular uptake, the three nanomaterials promoted the generation of excess ROS and mtROS. Interestingly, we found that ROS/mtROS levels were related to the amount of nanomaterials that entered the cells. This is partially because cobalt oxide particles are readily internalized via the endo-lysosomal pathway, and release of cobalt ions over long periods involves specific toxicity [40]. To assess the relationship between TNTs and ROS/mtROS, NAC and MitoQ were used to scavenge the ROS/mtROS after nanomaterials exposure. NAC and MitoQ reduced TNTs numbers and mitochondrial transfer, indicating that the ROS and mtROS produced by the nanomaterials were the main mechanism promoting TNTs formation and mitochondrial transfer. In conclusion, the difference in TNTs numbers upon nanomaterials exposure is mainly due to the different levels of ROS, whereas mtROS is a secondary factor in TNTs formation.

The PI3K/AKT/mTOR plays a key role in numerous cellular functions including proliferation, adhesion, migration, invasion, metabolism, and survival [41]. Importantly, we identified a new regulatory target of the PI3K/AKT/mTOR pathway in intercellular communication. In this study, three nanomaterials were found to activate the PI3K/AKT/mTOR pathway regardless of their properties. The activated pathway promotes TNTs formation and mitochondrial transfer. The LY294002, a broad-spectrum inhibitor of PI3K, PI3Kα, PI3Kδ and PI3Kβ [42], can inhibit the TNTs formation. Perifosine, a targeted Akt inhibitor [43], also reduced the TNTs number after nanomaterial treatment. However, nanomaterials-exposed groups were still higher than the control group, indicating that PI3K or AKT was not the only way to mediate TNTs formation. Interestingly, Rapamycin, an mTOR inhibitor, can potentially reduce TNTs formation, decreasing TNTs number to a basal level. These results indicated that mTOR plays a center role in TNTs formation. mTOR activates S6K1, which participates in mRNA translation, and then activates Eukaryotic Translation Elongation Factor 2 (EEF2) by phosphorylation. In addition, mTORC1 could deactivate the 4EBP1 protein which abolishes the inhibition of EIF4E, a transcription factor that aids in translation initiation by recruiting ribosomes to the 5'-cap structure [44]. EEF2 and EIF4E can bind to TNT-related genes, such as CDC42, to promote transcription. However, further investigation requires the identification of specific genes that play major roles in promoting TNTs following nanomaterial exposure.

The PI3K/AKT pathway is commonly downstream of ROS/mtROS and regulates ROS homeostasis for cell growth and proliferation. ROS can directly activate PI3K and inactivate phosphatase and tensin homolog, which negatively regulate the synthesis of PIP3 and then suppress AKT [45]. We found that NAC and MitoQ could reduce P110α and phosphorylated PI3K, and further reduce AKT levels. In addition, P110α can limit ROS/mtROS at a desirable range by promoting the cellular antioxidant mechanism via the NF-E2 p45-related factor 2-antioxidant response element dependent pathway [46].

**Fig. 9:**
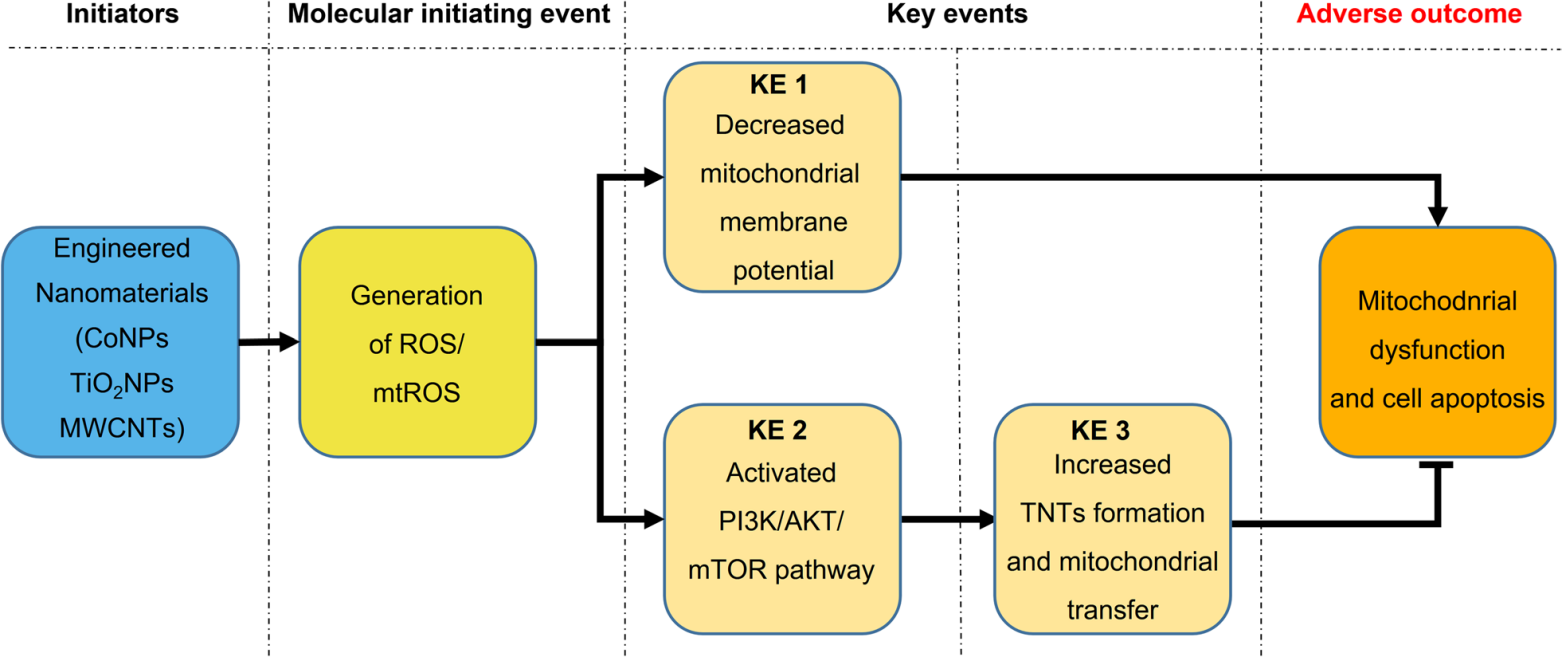
Proposed adverse outcome pathway for nanomaterials exposure. The scheme shows that the generation of cellular ROS and mitochondrial ROS induced by engineered nanomaterials is the molecular initiating event, resulting in decreased mitochondrial membrane potential (KE1), finally contributing to adverse outcomes of mitochondrial dysfunction and cell apoptosis. In addition, the molecular initiating event can activate the PI3K/AKT/mTOR pathway (KE2), which then promotes TNTs formation and mitochondrial transfer (KE3) against the adverse outcome

Adverse Outcome Pathway (AOP) concept provides a mechanism-based framework for interpreting what is known from existing toxicological studies of chemical substances, which covers the sequential progression of events from molecular initiation events (MIE) to adverse effects [47]. The main blocks of an AOP consist of the MIE, key events (KEs) as the mediators, and ultimately the ending, which is called adverse outcome (AO). Here, we summarized the AOP according to our findings to promote a better understanding of the role of TNTs induced by nanomaterials in neurotoxicity (Fig. 9). The generation of ROS and mtROS induced by engineered nanomaterials is the molecular initiating event, which subsequently decreases mitochondrial membrane potential (KE1), thus leading to adverse outcomes of mitochondrial dysfunction and cell apoptosis. In addition, MIE can activate the PI3K/AKT/mTOR pathway (KE2), which then promotes TNTs formation and mitochondrial transfer (KE3) against the adverse outcome.

## Conclusion

This is the first study to unveil that different types of engineered nanomaterials induce the formation of TNTs in human glial cells to protect against neurotoxicity via ROS/mtROS-centered activation and the downstream PI3K/AKT/mTOR pathway. Despite their different biophysical properties, three types of nanomaterials, namely CoNPs, TiO_2_NPs and MWCNTs, activate TNTs-dependent mitochondrial transfer in primary astrocyte and U251 cells, which can rescue mitochondrial damage and cell apoptosis caused by oxidative stress. Most importantly, the adverse outcome pathway was summarized to shed light on the intercellular protection mechanism against nanomaterials-induced neurotoxicity.

### Supplementary Information

**Additional file 1:** Supporting information of ROS/mtROS promotes TNTs formation via the PI3K/AKT/mTOR pathway to protect against mitochondrial damages in glial cells induced by engineered nanomaterials.** Fig. S1.** TNTs numbers in SH-SY5Y and U251 cells under physiological condition.** Fig. S2.** Nanomaterials decreased the viability of U251 cells. **Fig. S3.** Characterization of PA.** Fig. S4.** Three nanomaterials’ autofluorescence did not interfere with the detection of ROS and mtROS.** Fig. S5.** The concentration of endotoxin in 1 mg/mL nanomaterials.** Fig. S6.** TNTs restores mitochondrial damage and apoptosis induced by nanomaterials in U251 cells.** Fig. S7.** NAC and MitoQ alleviate the ROS level induced by nanomaterials in U251 cells.

**Additional file 2: Video S1.** Dynamic observation of vesicle transfer between astrocytes upon physical condition. Vesicles were marked by DiD probe (represented as Red color). Scale bar = 25 μm.

**Additional file 3: Video S2.** Dynamic observation of vesicle transfer between astrocytes upon CoNPs exposure. Vesicles were marked by DiD probe (represented as Red color). Scale bar = 25 μm.

**Additional file 4: Video S3.** Dynamic observation of vesicle transfer between astrocytes upon TiO_2_NPs exposure. Vesicles were marked by DiD probe (represented as Red color). Scale bar = 25 μm.

**Additional file 5: Video S4.** Dynamic observation of vesicle transfer between astrocytes upon MWCNTs exposure. Vesicles were marked by DiD probe (represented as Red color). Scale bar = 25 μm.

**Additional file 6: Video S5.** Dynamic observation of mitochondrial transfer between astrocytes upon physical condition. Mitochondria were labeled by MitoTrakcer (represented as Yellow color). Scale bar = 25 μm.

**Additional file 7: Video S6.** Dynamic observation of mitochondrial transfer between astrocytes upon CoNPs exposure. Mitochondria were labeled by MitoTrakcer (represented as Yellow color). Scale bar = 25 μm.

**Additional file 8: Video S7.** Dynamic observation of mitochondrial transfer between astrocytes upon TiO_2_NPs exposure. Mitochondria were labeled by MitoTrakcer (represented as Yellow color). Scale bar = 25 μm.

**Additional file 9: Video S8.** Dynamic observation of mitochondrial transfer between astrocytes upon MWCNTs exposure. Mitochondria were labeled by MitoTrakcer (represented as Yellow color). Scale bar = 25 μm.

**Additional file 10:** Dynamic observation of uptake of TiO_2_NPs in U251 cells in bright field. Scale bar = 25 μm.

**Additional file 11:** Origin, full-length gels and blot images.

## Data Availability

All data analyzed within this study are included either in the manuscript or in the additional files.

## Abbreviations

AKT: Protein kinase B
ANOVA: One-way analysis of variance
AO: Adverse outcome
AOP: Adverse outcome pathway
CAGR: Compound annual growth rate
CNS: Central nervous system
CoNPs: Cobalt nanoparticles
EEF2: Eukaryotic translation elongation factor 2
EVs: Extracellular vesicles
FBS: Fetal bovine serum
FSC: Forward-scattered light
HCS: High content screening system
KE: Key events
LAL: Limulus amebocyte lysate
LAT-B: Latrunculin B
MIE: Molecular initiation events
MitoQ: Mitoquinone
MMP: Mitochondrial membrane potential
mTOR: Mammalian target of rapamycin
mtROS: Mitochondrial reactive oxygen species
MWCNTs: Multi-walled carbon nanotubes
NAC: N-acetyl-L-cysteine
PA: Primary astrocyte
PBS: Phosphate-buffered saline
PI3K: Phosphatidylinositol 3-kinase
ROS: Reactive oxygen species
RT: Room temperature
SDS: Sodium dodecyl sulfate
SEM: Scanning electron microscope
SSC: Side-scattered light
TBST: Tris-buffered saline containing Tween-20
TEM: Transmission electron microscopy
TiO_2_NPs: Titanium dioxide nanomaterials
TNTs: Tunneling nanotubes
