# Supplementary material for: ROS/mtROS promotes TNTs formation via the PI3K/AKT/mTOR pathway to protect against mitochondrial damages in glial cells induced by engineered nanomaterials
Source: Part Fibre Toxicol. 2024 Jan 15;21:1. doi: 10.1186/s12989-024-00562-0 (PMC10789074; doi:10.1186/s12989-024-00562-0)
Supplement: Supplementary file 1 — Additional file 1: Supporting information of ROS/mtROS promotes TNTs formation via the PI3K/AKT/mTOR pathway to protect against mitochondrial damages in glial cells induced by engineered nanomaterials. Fig. S1. TNTs numbers in SH-SY5Y and U251 cells under physiological condition. Fig. S2. Nanomaterials decreased the viability of U251 cells. Fig. S3. Characterization of PA. Fig. S4. Three nanomaterials’ autofluorescence did not interfere with the detection of ROS and mtROS. Fig. S5. The concentration of endotoxin in 1 mg/mL nanomaterials. Fig. S6. TNTs restores mitochondrial damage and apoptosis induced by nanomaterials in U251 cells. Fig. S7. NAC and MitoQ alleviate the ROS level induced by nanomaterials in U251 cells. [file 12989_2024_562_MOESM1_ESM.docx]

**Supporting information for**

**ROS/mtROS promotes TNTs formation via the PI3K/AKT/mTOR pathway to protect against mitochondrial damages in glial cells induced by engineered nanomaterials**

Xinpei Lin^a 1 2 3^, Wei Wang^a 1 2 3^, Xiangyu Chang^a 1 2 3^, Cheng Chen ^1 3^, Zhenkun Guo^1 3^, Guangxia Yu^1 3^, Wenya Shao^1 3^, Siying Wu ^3 4^, Qunwei Zhang ^5^, Fuli Zheng* ^1 2 3^, Huangyuan Li * ^1 3^

1 Department of Preventive Medicine, School of Public Health, Fujian Medical University, Fuzhou, 350122, Fujian Province, China

2 Fujian Provincial Key Laboratory of Molecular Neurology, Institute of Neuroscience, Fujian Medical University, 350004, Fuzhou, China

3 The key Laboratory of Environment and Health, School of Public Health, Fujian Medical University, Fuzhou, 350122, Fujian Province, China

4 Department of Epidemiology and Health Statistics, School of Public Health, Fujian Medical University, Fuzhou, 350122, Fujian Province, China

5 Department of Epidemiology and Population Health, School of Public Health and Information Sciences, University of Louisville, 485 E. Gray Street, Louisville, USA

* Correspondence: H.L: lhy@fjmu.edu.cn; F. Z.: f.zheng@fjmu.edu.cn

**Additional file 1**


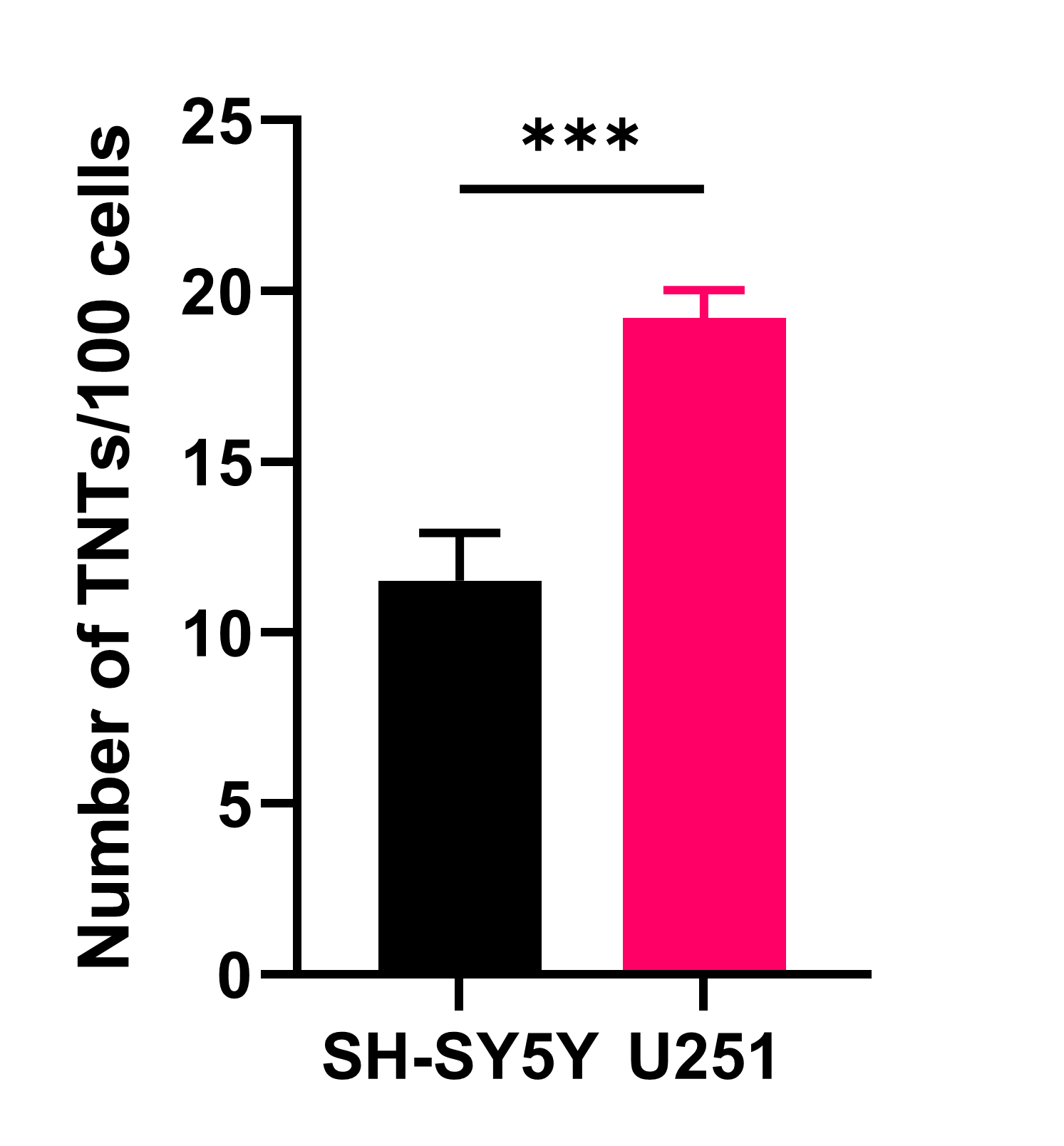


**Fig. S1. TNTs numbers in SH-SY5Y and U251 cell under physiological condition.** Quantification of TNTs number. *** *P* < 0.01. Date present as mean ± SEM. n=3.


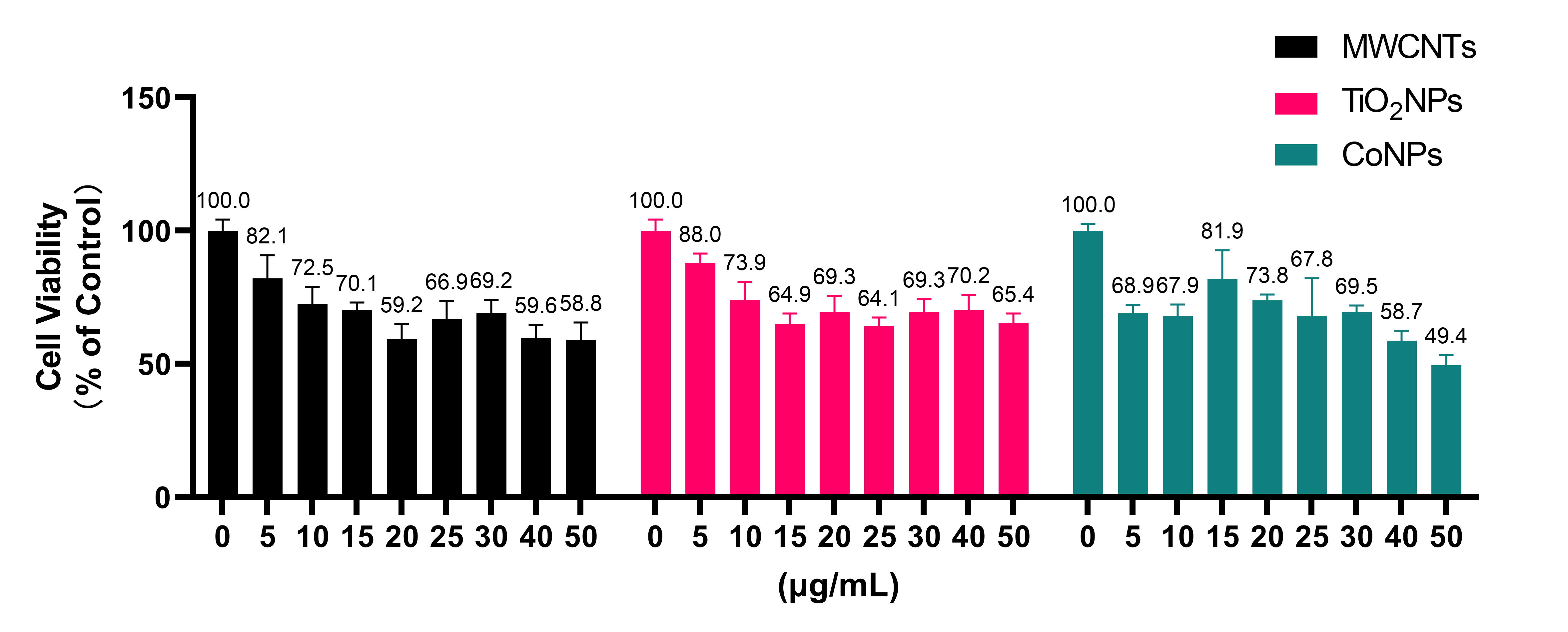


**Fig. S2. Nanomaterials decreased the viability of U251 cells.** U251 cells were exposed to different concentrations of three types of nanomaterials (0, 5, 10, 15, 20, 25, 30, 40, and 50 μg/mL) for 24 h. CCK8 was used to check the cell viability. Date present as mean ± SEM. n=6.


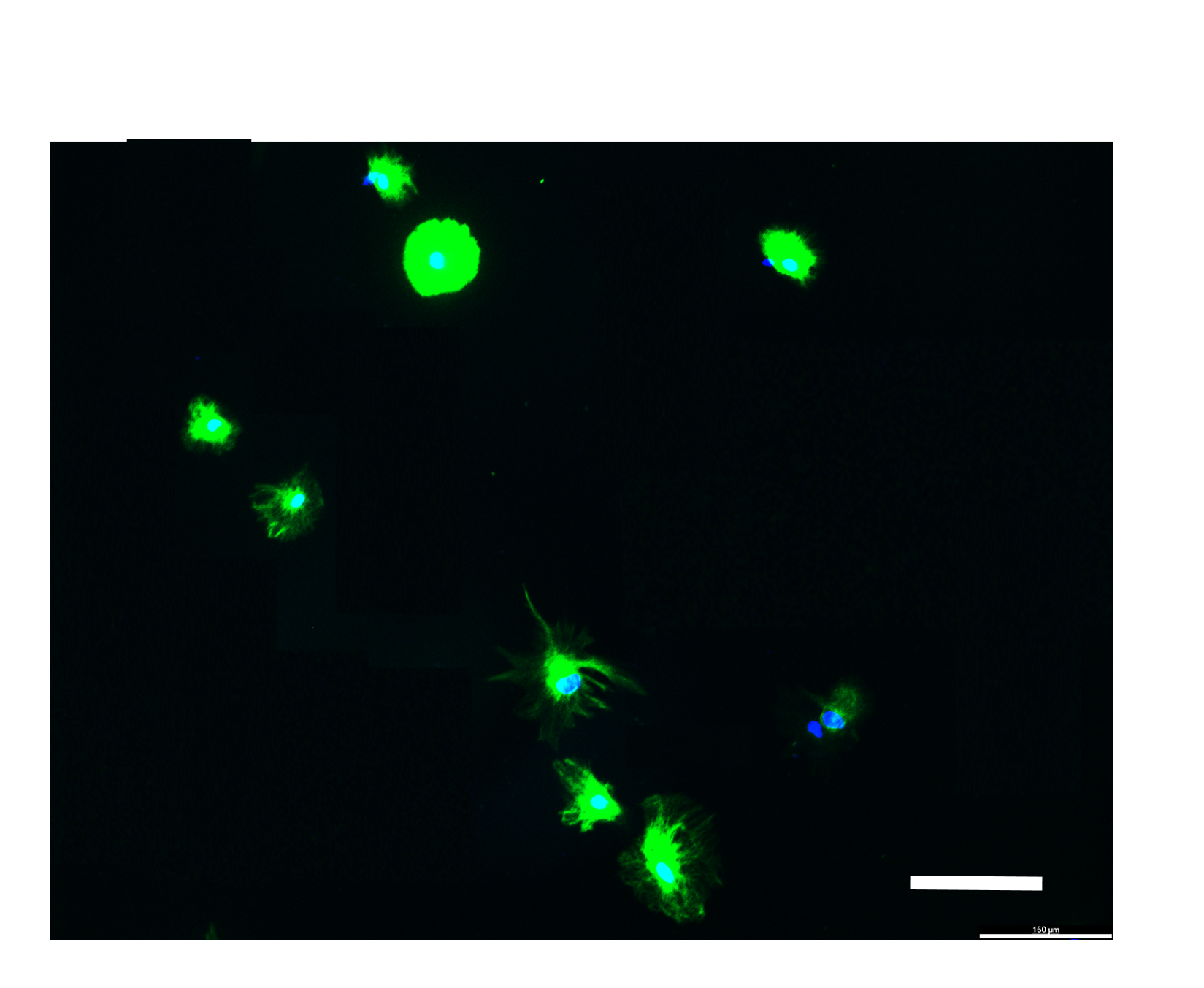


**Fig. S3. Characterization of PA.** Representative image of GFAP-positive cells. n=3. Scale bar = 100 μm.


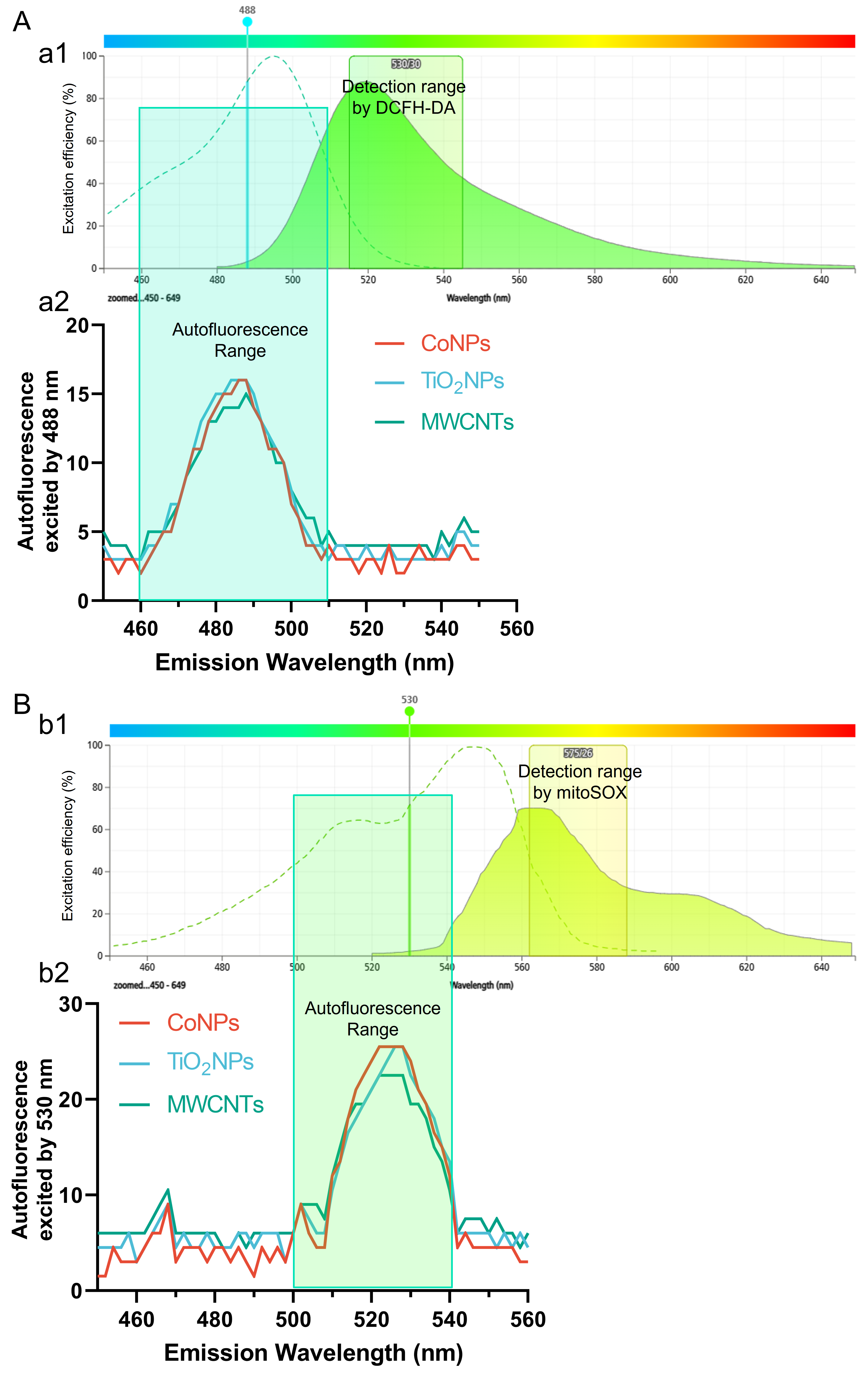


**Fig. S4. Three nanomaterials’ autofluorescence did not interfere with the detection of ROS and mtROS. (A)** The emission spectrum of DCFH-DA did not overlap with the emission spectrum of three nanomaterials under 488 nm excitation wavelength. **(a1)** A light blue dashed curve represents the excitation spectrum of DCFH-DA and a curve filled with green indicates the emission spectrum of DCFH-DA. The light blue dashed line represents the excitation wavelength (515-545 nm) of the microscope (DMi8, Leica), and the light green box indicates the detection wavelength of the microscope. **(a2)** The emission wavelength (460-510 nm) of three nanomaterials excited by 488 nm (also excited DCFH-DA) was measured by Infinite 200 PRO (Tecan Trading AG, Switzerland). **(B)** The emission spectrum of mitoSOX did not overlap with the emission spectrum of three nanomaterials under 530 nm excitation wavelength. **(b1)** Yellow green curve represents the excitation spectrum of mitoSOX and the curve filled yellow indicates the emission spectrum of mitoSOX. The yellow-green line represents the excitation wavelength of the microscope and the yellow box indicates the detection wavelength (568-588 nm) of the microscope. **(b2)** The emission wavelength (510-540 nm) of three nanomaterials excited by 530 nm (also excited mitoSOX) was measured by Infinite 200 PRO.

**Method in detail:** To rule out the interference of nanomaterials’ autofluorescence on ROS and mtROS, we have conducted a series of experiments. First, we checked the excitation and emission spectrum of the DCFH-DA and mitoSOX probe, as shown in Fig 1A (a1, b1). When DCFH-DA is detected by the fluorescence microscope (DMi8, Leica), the 488 nm excitation wavelength is used to excite the DCFH-DA, and the detection wavelength is 515-545 nm (the light green box). As for mitoSOX probe detection, the 530 nm excitation wavelength and the detection wavelength (568-588 nm) were used to measure it using the microscope. Then, we detected the emission spectrum of three nanomaterials under the same excitation wavelength (Fig 1, a2, b2). Under the 488 nm excitation wavelength, the emission spectrum of nanomaterials is 460-500 nm, which did not overlap with the microscope’s detection wavelength (515-545 nm). Under 530 nm excitation wavelength, the emission spectrum of three nanomaterials is 510-540 nm, which did not overlap with the microscope’s detection wavelength (568-588 nm). Therefore, we can exclude the interference of nanomaterials’ autofluorescence on the DCFH-DA and mitoSOX probe.


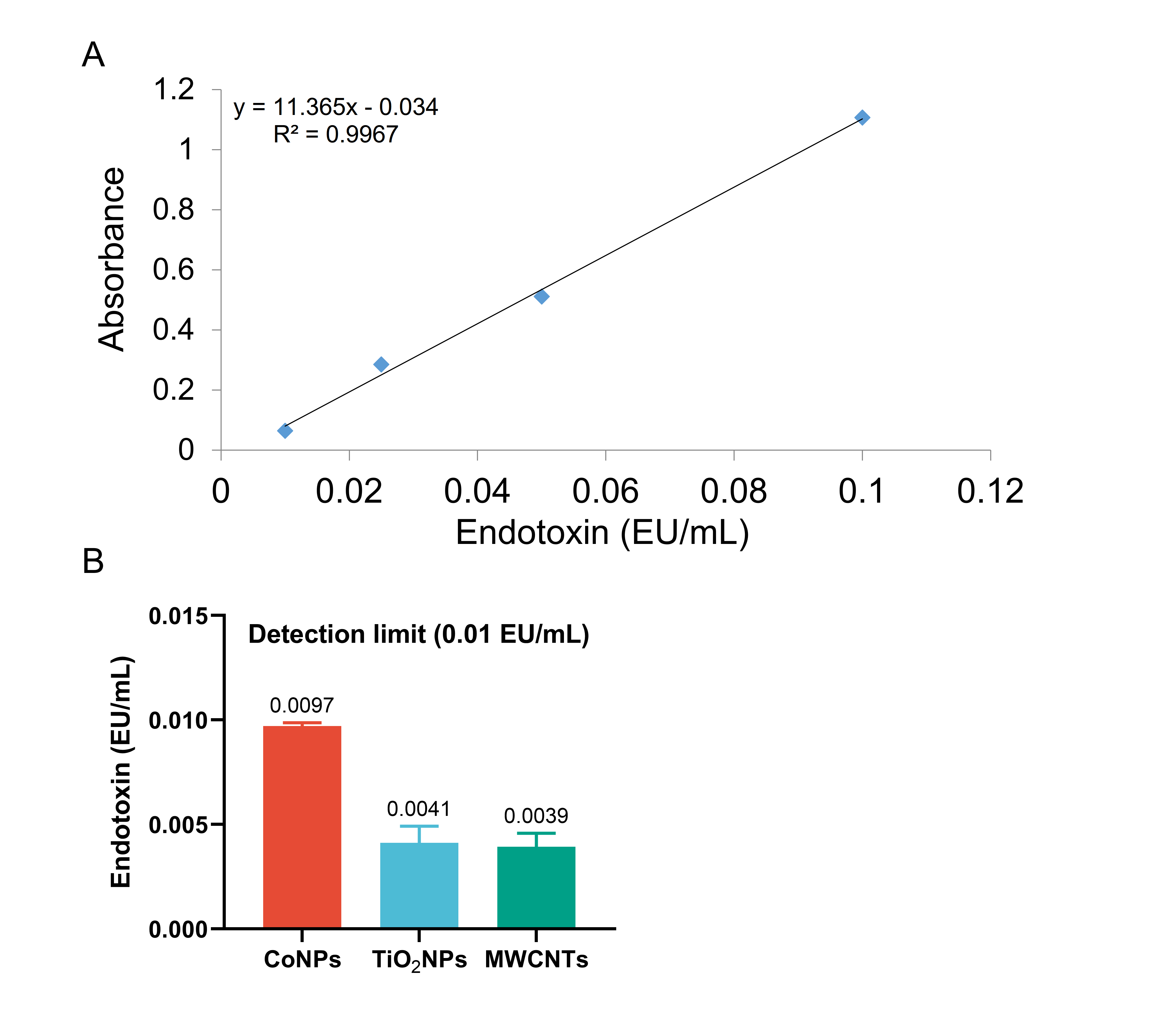


**Fig. S5. The concentration of endotoxin in 1 mg/mL nanomaterials. (A)** The standard curve was drawn according to the absorbance of different concentrations of endotoxin standard substances (0.01, 0.025, 0.05, 0.1 EU/mL). **(B)** The endotoxin content of three nanomaterials. n = 3.

**Method in detail:** Endotoxin was measured by the endpoint chromogenic Limulus Amebocyte lysate (LAL) assay kit (EC32545, BIOENDO, Xiamen, China). Briefly, the standard curve was drawn according to the absorbance of different concentrations of endotoxin standard substances (0.01, 0.025, 0.05, 0.1 EU/mL), and then the content of endotoxin of nanomaterials was calculated according to the absorbance of measured samples.


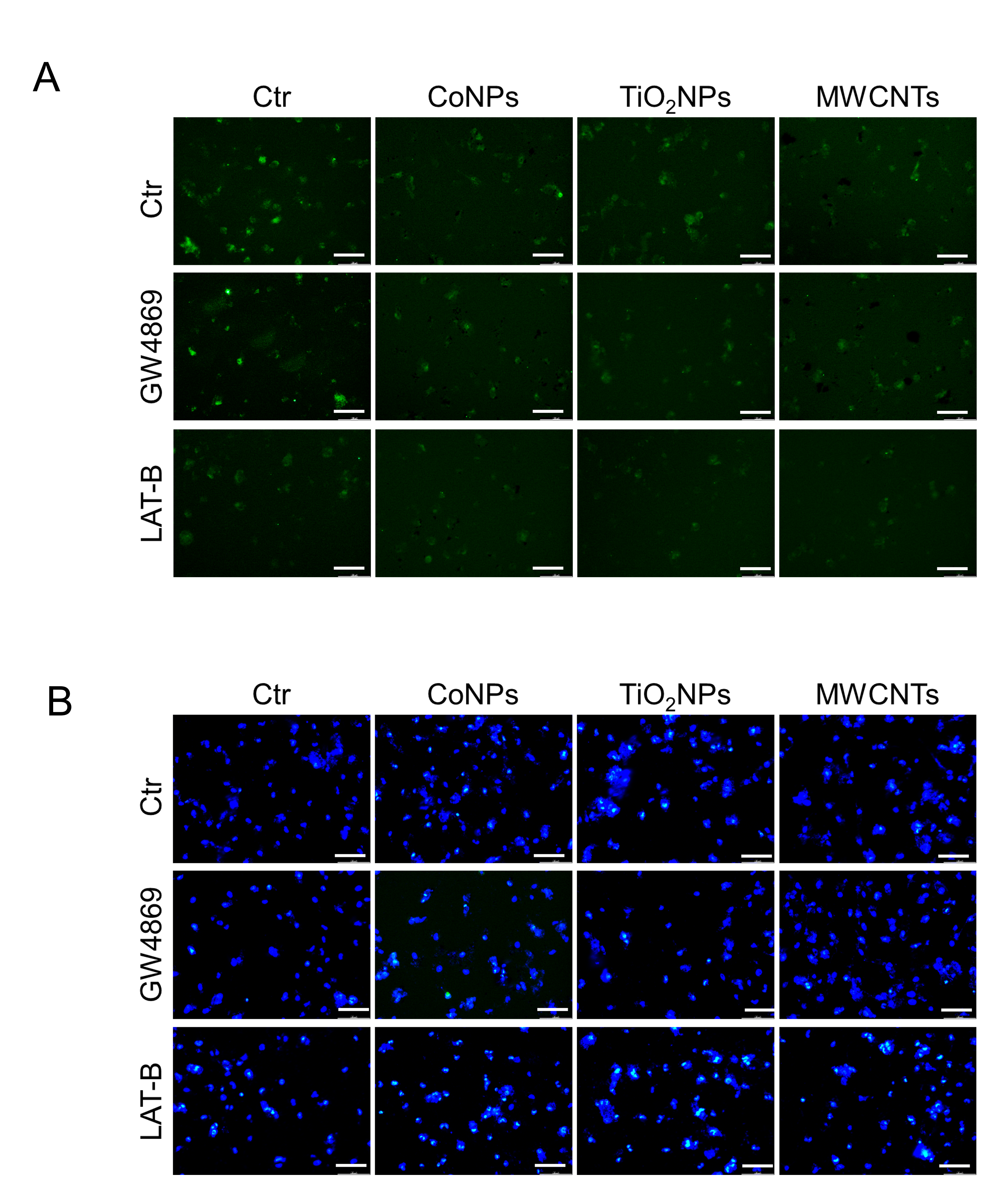


**Fig. S6. TNTs restores mitochondrial damage and apoptosis induced by nanomaterials in U251 cells. (A)** Representative image of the level of ATP in U251 cells. **(B)** Representative image of apoptosis in U251 cells. Scale bar = 100 μm. n=3.


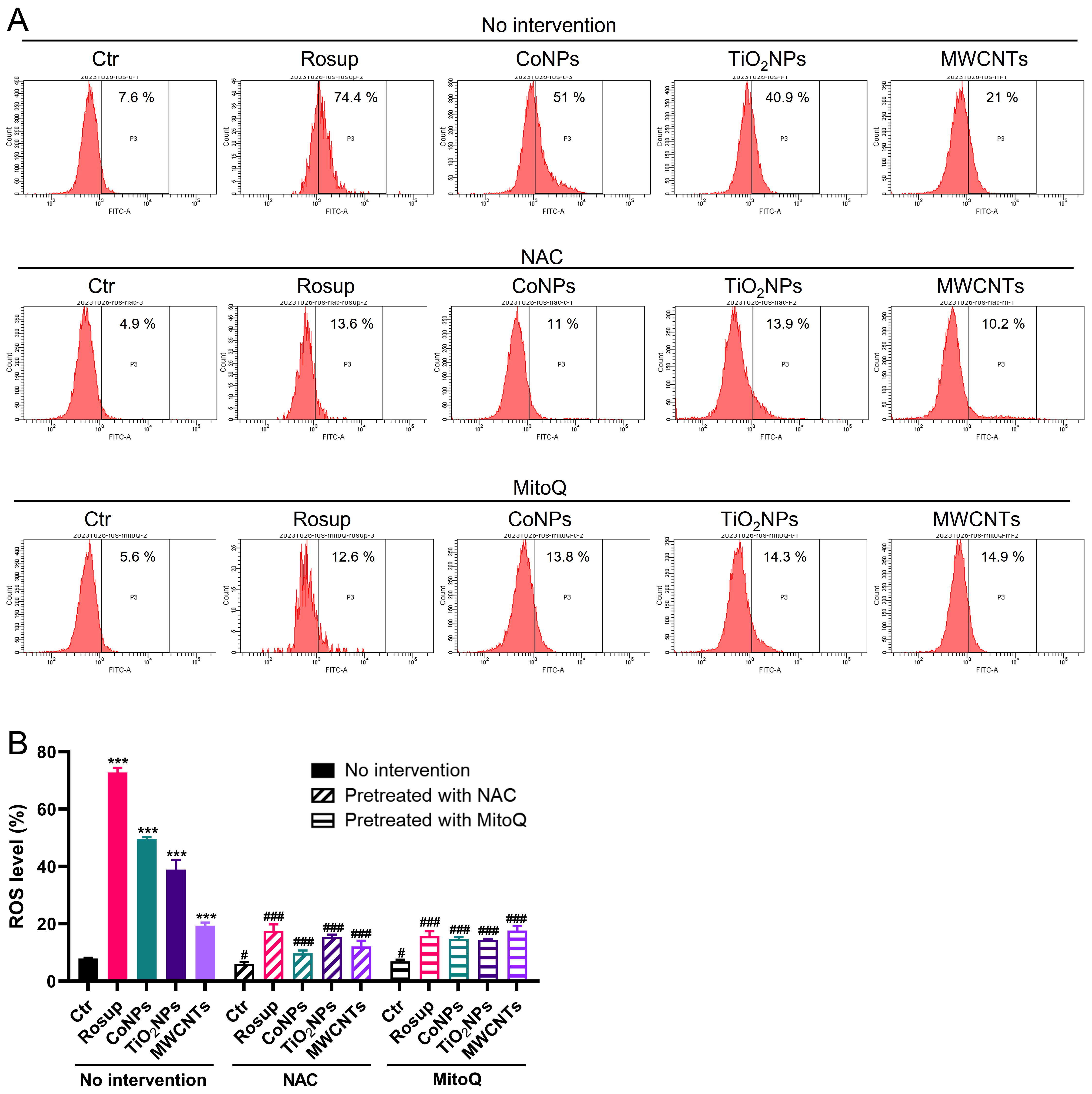


**Fig. S7. NAC and MitoQ alleviate the ROS level induced by nanomaterials in U251 cells.** Cells were pretreated with 10 mM NAC for 30 min and then exposed to nanomaterials for 24 h. Rosup, ROS positive control, was added to the plate with a final concentration (250 μg/mL) for 30 min. And then U251 cells were incubated with 1 μM DCFH-DA at 37°C for 30 min. The level of ROS analyzed by flow cytometry at an excitation wavelength of 488 nm and an emission wavelength of 525 nm. For each sample, 10000 events were recorded. **(A)** Representative image of ROS level measured by cytometry in U251 cells. **(B)** Statistic analysis of ROS level in U251 cells. Rosup: ROS inducer. *** *P* < 0.001 compared with the no intervention control group. ^#^ and ^###^ *P* < 0.05 and 0.001 compared with the respective nanomaterials group (regarding the no intervention nanomaterials group). Date present as mean ± SEM. n=3.
