# Supplementary material for: ROS/mtROS promotes TNTs formation via the PI3K/AKT/mTOR pathway to protect against mitochondrial damages in glial cells induced by engineered nanomaterials
Source: Part Fibre Toxicol. 2024 Jan 15;21:1. doi: 10.1186/s12989-024-00562-0 (PMC10789074; doi:10.1186/s12989-024-00562-0)

Origin, full-length gels and blot images of Figure 7

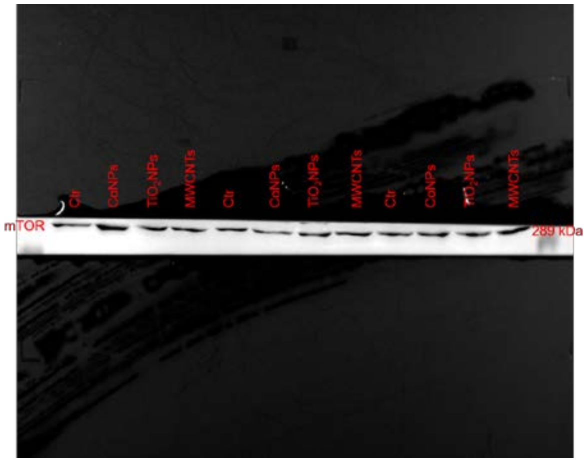

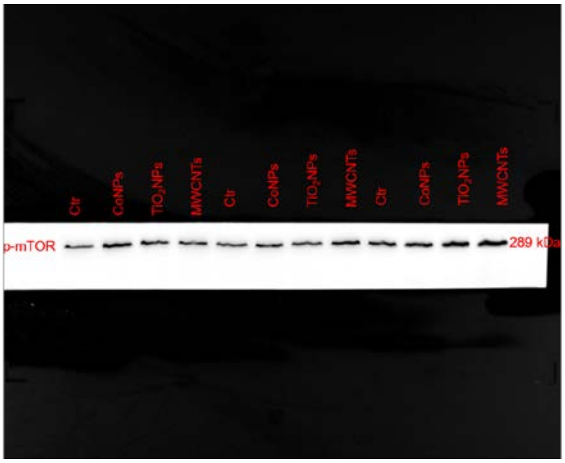

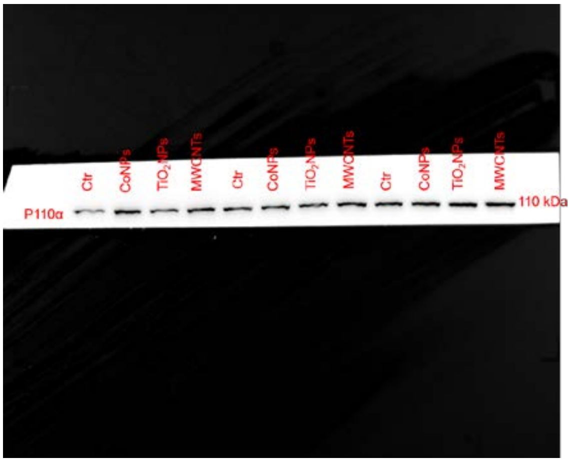

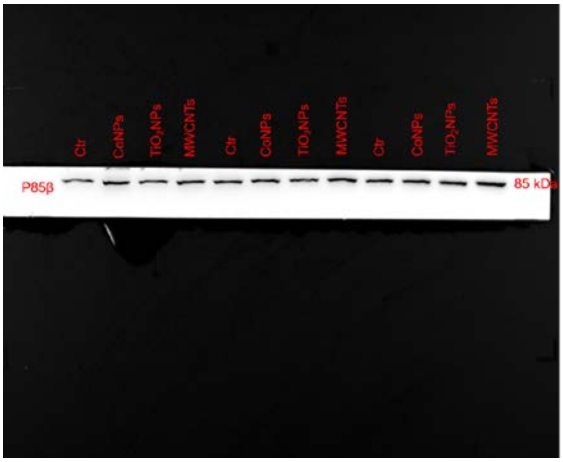

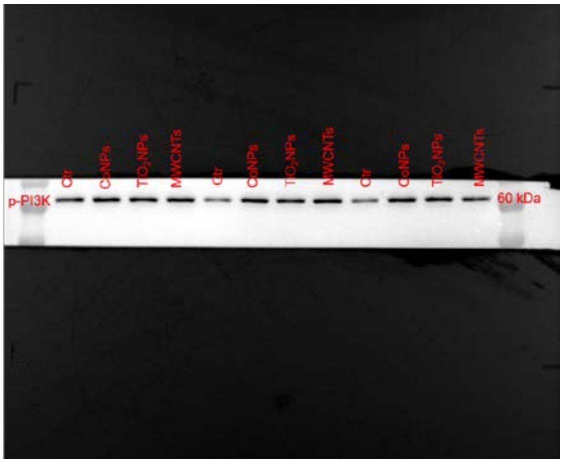

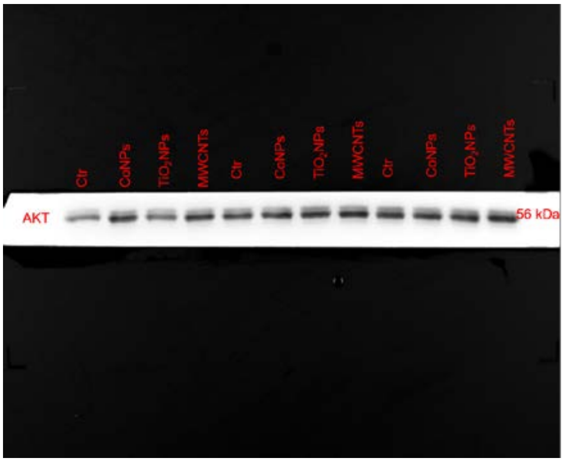

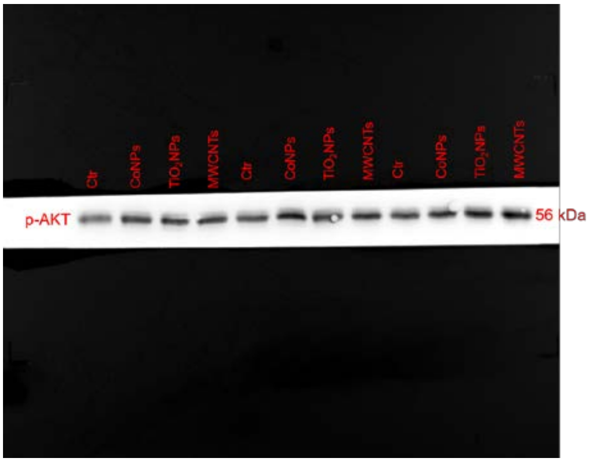

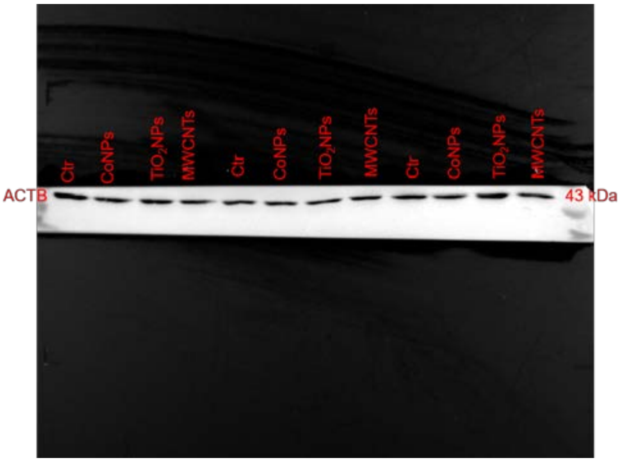

Origin, full-length gels and blot images of Figure8

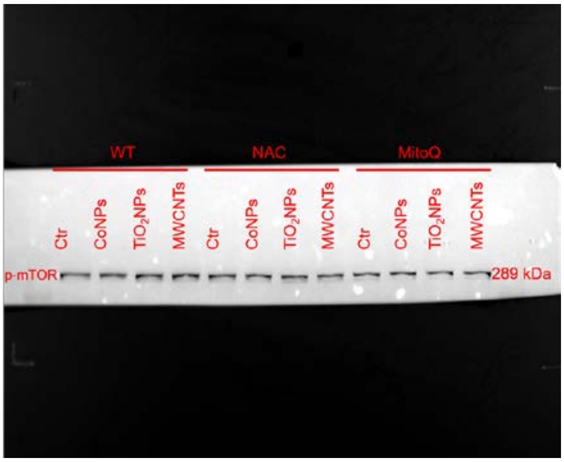

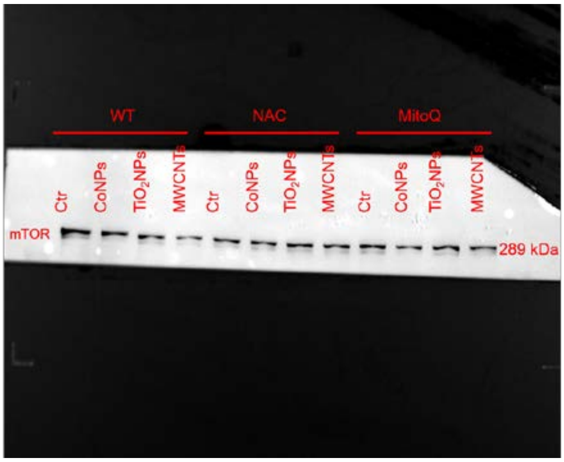

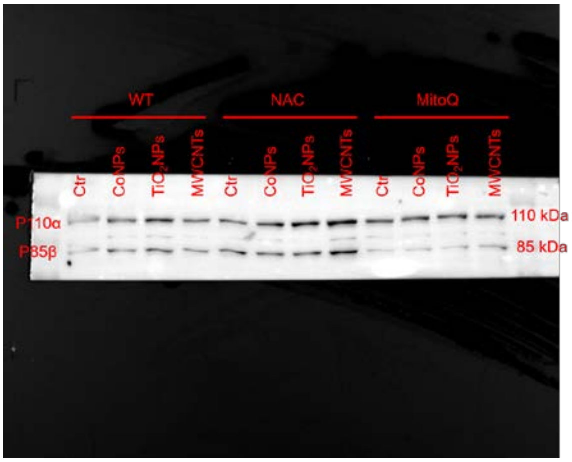

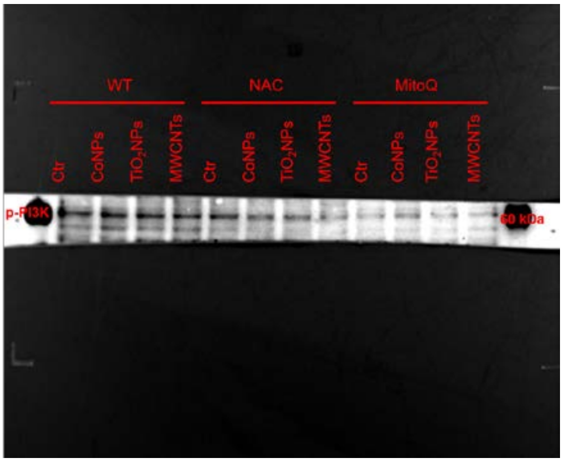

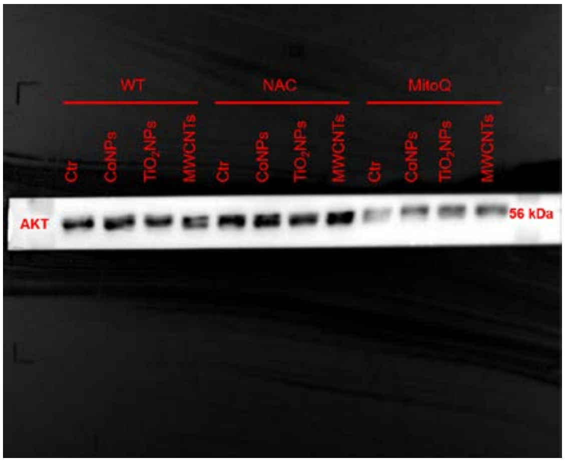

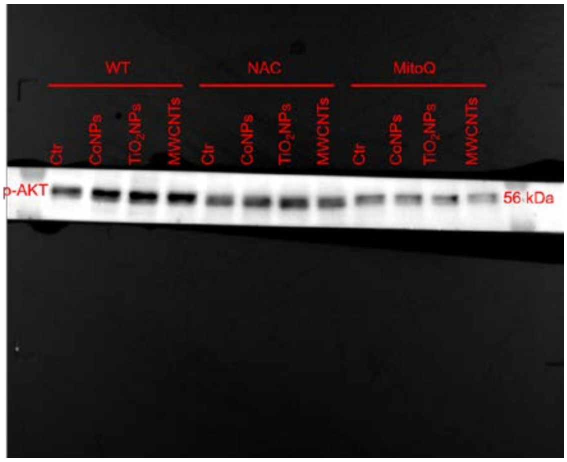

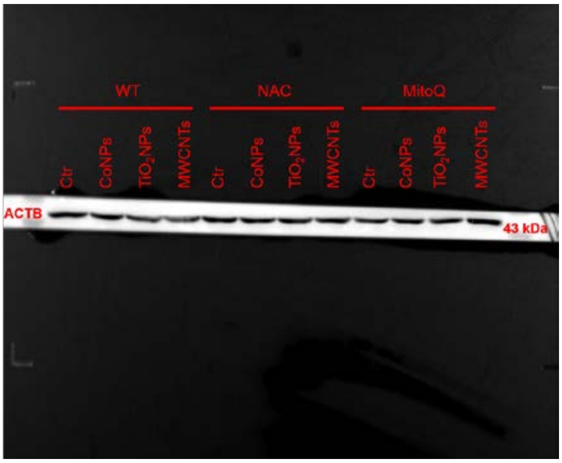

Supplement: Supplementary file 11 — Additional file 11: Origin, full-length gels and blot images. [file 12989_2024_562_MOESM11_ESM.pdf]
